# Supplementary material for: Efficient and accurate search in petabase-scale sequence repositories
Source: Nature. 2025 Oct 8;647(8091):1036–44. doi: 10.1038/s41586-025-09603-w (PMC12657231; doi:10.1038/s41586-025-09603-w)
Supplement: Supplementary file 1 — Supplementary Methods, Supplementary Figs. 1–17, Supplementary Tables 1–4 and Supplementary References. The Supplementary Methods detail the data structures and algorithms used for constructing, annotating and querying MetaGraph indexes. The Supplementary Figures contain explanatory figures for our data structures and algorithms, benchmarking experiments for our software and additional evaluations of or with MetaGraph indexes. The Supplementary Tables describe the 100-study index constructed from sequencing reads. [file 41586_2025_9603_MOESM1_ESM.pdf]

nature portfolio

https://doi.org/10.1038/s41586-025-09603-w

Supplementary information

Efficient and accurate search in petabase-scale sequence repositories

In the format provided by the authors and unedited

Supplementary Material

Efficient and Accurate Search in Petabase-Scale Sequence Repositories

Mikhail Karasikov,[1,2,3,*] Harun Mustafa,[1,2,3,*] Daniel Danciu,[1,2] Oleksandr Kulkov,[1,2] Marc Zimmermann,[1,2] Christopher Barber,[1,2] Gunnar Rätsch,[1,2,3,4,5,‡] and André Kahles[1,2,3,‡]

[1]Biomedical Informatics Group, Department of Computer Science, ETH Zurich, Zurich, Switzerland
[2]Swiss Institute of Bioinformatics, Zurich, Switzerland
[3]Medical Informatics, University Hospital Zurich, Zurich, Switzerland
[4]Department of Biology, ETH Zurich, Zurich, Switzerland
[5]AI Center, ETH Zurich, Zurich, Switzerland

[*]Equal contribution. [‡]To whom correspondence should be addressed.

Contents

A Supplementary Methods **5**
- A.1 Notation . . . 5
- A.2 Basic building blocks: compressed bitmap representations . . . 5
  - A.2.1 Schemes for the compressed representation of bit vectors . . . 6
  - A.2.2 Benchmarks and hybrid bit vector representations . . . 7
- A.3 Indexing sequences in de Bruijn graphs . . . 7
  - A.3.1 Representing a de Bruijn graph . . . 9
    - Hash table-based de Bruijn graph representations . . . 9
    - Representing a k-mer dictionary with an indicator bitmap . . . 9
  - A.3.2 Succinct k-mer dictionaries represented as a BOSS table . . . 10
    - The BOSS representation . . . 10
    - k-mer traversals . . . 11
    - Decoding k-mers . . . 12
    - k-mer lookups . . . 13
    - Indexing k-mer ranges by suffix . . . 13
    - Dummy k-mers in the BOSS table . . . 14
    - Internal representations of the BOSS table . . . 14
  - A.3.3 Graph construction . . . 15
    - Distributed construction . . . 15
- A.4 Representing binary matrices in MetaGraph . . . 16
  - A.4.1 Column-major matrix representations . . . 16
  - A.4.2 Row-major matrix representations . . . 16
  - A.4.3 The Rainbow matrix decomposition technique . . . 17
- A.5 Estimating the cost of comprehensive nucleotide search engine . . . 17
  - A.5.1 General estimation methodology and assumptions for MetaGraph . . . 17
  - A.5.2 Hosting scenarios . . . 18

B Supplementary Figures **19**

C Supplementary Tables **29**

Supplementary References **29**

Chapter A

Supplementary Methods

A.1 Notation

Let Σ be an alphabet of fixed size (e.g., $\{\mathsf{A}, \mathsf{C}, \mathsf{G}, \mathsf{T}, \mathsf{N}\}$ for DNA sequences and $\{\mathsf{A}, \mathsf{R}, \mathsf{N}, \ldots, \mathsf{V}, \mathsf{X}\}$ for amino acid sequences). Given a choice of alphabet Σ, we define the extended alphabet $\hat{\Sigma} = \Sigma \cup \{\$\}$ extended by the sentinel character $\$$, which is lexicographically smaller than every character of Σ.

Given a string s, let $|s|$ denote its length and let ε denote the empty string (i.e., $|\varepsilon| = 0$). Let s_i denote the i-th character of s and $s_{i:j}$ denote the substring $s_i \cdots s_j$), where $1 \leq i \leq j \leq |s|$. We additionally use the notation $s_{:j}$ to denote the prefix $s_{1:j}$ and $s_{i:}$ to denote the suffix $s_{i:|s|}$. Given another string s' of length m', we denote its concatenation with s by $ss' = s_1 \cdots s_{|s|}\, s'_1 \cdots s'_{|s'|}$. The power function c^k of a character (string) c defines the concatenation of k characters (strings) c. Lexicographical order on strings is denoted by $<$, and co-lexicographical order (i.e., lexicographical order on the reversed strings) by $<^{\text{colex}}$.

Given a positive number k, every string of length k is called a *k-mer*. Every k-mer over the initial alphabet Σ is called a *real k-mer* and every k-mer containing at least one sentinel character $\$$ from the extended alphabet $\hat{\Sigma}$ is called a *dummy k-mer*.

In addition, we use functions $\text{rank}_\cdot(\cdot,\cdot)$ and $\text{select}_\cdot(\cdot,\cdot)$ defined for strings in arbitrary alphabets as follows. For a given string s, a character c, and a positive index $i \geq 1$, function $\text{rank}_c(s, i)$ returns the number of characters c occurring in the string s before index i,

$$\text{rank}_c(s, i) = \#\{j \leq i \mid s_j = c\}, \tag{A.1}$$

and function $\text{select}_c(s, i)$ returns the index of the i-th occurrence of character c in s,

$$\text{select}_c(s, i) = \min\{j \mid \text{rank}_c(s, j) = i\} \tag{A.2}$$

if $i \leq \text{rank}_c(s, |s|)$, and undefined otherwise. For arbitrary arrays and vectors, the functions $\text{rank}_\cdot(\cdot,\cdot)$ and $\text{select}_\cdot(\cdot,\cdot)$ are defined similarly as for strings.

A.2 Basic building blocks: compressed bitmap representations

In order to minimize the space taken by the MetaGraph indexes while still supporting fast queries, we make heavy use of *compressed data structures*. In this section, we will describe different basic data structures used in MetaGraph for representing bit vectors, which are instrumental in building

more complex data structures, such as those encoding graphs and matrices in the MetaGraph index.

A.2.1 Schemes for the compressed representation of bit vectors

Static bit vector. The fastest of the representations considered here packs the bits in an array of 8-byte integers (stored in type `uint64_t`). In this representation, which we refer to as stat, the data is stored uncompressed and therefore takes $n + O(1)$ bits of space, but each bit can be queried in $O(1)$ time, and hence, very quickly in practice. In particular, we used the class `sdsl::bit_vector` from the *sdsl-lite* library [91]. To enable *rank* and *select* operations, two additional data structures have to be added. For these, we used `sdsl::rank_support_v5` and `sdsl::select_support_mcl` data structures from the *sdsl-lite* library, respectively.

`sdsl::rank_support_v5` splits the array into 2048-bit blocks (called *superblocks*) and stores precomputed *rank* values for the last position of each block. This requires 64/2048 bits of space per each represented bit. On top of that, it subdivides each superblock into 5 blocks where the relative rank values for each of them are fitted into another 64-bit word as $5 \log_2 2048 = 55 < 64$. Thus, this additional structure requires an extra $128/2048 = 6.25\%$ of additional space while reducing the time complexity of rank_1 to $O(1)$, and more precisely, a few word accesses and popcounts.

`sdsl::select_support_mcl` stores positions of every 4096th set bit in an auxiliary vector. These bits are called *sampled bits.* If the distance between a pair of consecutive sampled bits is greater than $\log_2^4 n$, each of the 4096 positions of set bits between them are stored in a packed array using $\log_2 n$ bits per position. This takes at most $\frac{4096 \cdot \log_2 n}{\log_2^4 n} = 4096/\log_2^3 n$ bits per entry. If the distance between a pair of consecutive sampled bits is less than $\log_2^4 n$, it explicitly stores only the relative positions of every 64-th set bit using less than $\log_2 \log_2^4 n = 4 \log_2 \log_2 n$ bits. This takes, in total, less than $\frac{4 \log_2 \log_2 n}{64}$ bits per bit.

Note that the auxiliary `sdsl::rank_support_v5` and `sdsl::select_support_mcl` data structures are optional and are initialized only when the respective *rank* or *select* operations have to be supported.

Compressed bit vector sdarray. The sdarray compressed data structure represents very sparse bit vectors. It stores the positions of set bits using the Elias-Fano encoding for non-decreasing sequences. The $w := \max(1, \lceil \log_2 n \rceil - \lceil \log_2 m \rceil)$ least significant bits of each position are stored in a packed integer vector, where n is the size of the bit vector and m is the number of set bits in it. Hence, the least significant bits take $mw + O(1)$ bits of space. The remaining most significant bits are represented with a delta coding taking $m + \lfloor n/2^w \rfloor + O(1)$ bits. After simplifications, the total representation size is $m(2 + \log_2 \frac{n}{m})(1 + o(1))$ bits, plus the overhead from two `sdsl::select_support_mcl` data structures enabling the select_1 operation in $O(1)$ time, as well as the rank_1 and the access operations in $O(\log \frac{n}{m})$ time. In practice, we used the `sdsl::sd_vector` class from the *sdsl-lite* library [91]. Since this representation was specifically designed for sparse bit vectors and generates an unreasonably large overhead when representing dense vectors, we flip the bits of the represented bitmap when its density is greater than 0.5.

RRR succinct bit vector. Next, we used RRR vectors to provide excellent time performance for *rank* and *select* queries in practice. We used the implementation of the RRR scheme from the *sdsl-lite* library [91]. One notable parameter of RRR vectors is the block size, which provides a practical trade-off between speed and space overhead. As a result of our preliminary benchmarks, we decided to restrict the choice of available block sizes to two values: 15 and 63, as the improvement of query times for other values comes at too high a cost of the extra space overhead and vice versa.

Dynamic bit vector. To support dynamic insertions and deletions, in addition to access, rank, and select queries, we represent bit vectors as B-trees with $B = 16$ children per internal node and each leaf storing a respective 8192-bit block of the represented bitmap [92]. This representation naturally supports insertions and deletions in $O(\log n)$ time.

A.2.2 Benchmarks and hybrid bit vector representations

We measured the representation size (**Supplementary Figure S-1**) and query speed (**Supplementary Figure S-2**) for each scheme. For this experiment, we generated a series of bit vectors of size 10^9 and different densities with uniformly distributed set bits. It can be seen that, depending on the density, each scheme has a region where it performs best, either in speed or representation size. Thus, we decided to add three hybrid schemes that would switch between some of the base representation schemes described above, depending on the density of the represented bitmap and the desired query performance and space complexity.

The first scheme is called smart and switches between stat and sdarray (sd) representations to provide a very good query performance and, at the same time, take advantage of sparsity and ensure a very good compression for sparse bitmaps.

Next, the small representation employs the RRR scheme with a block size of 63 except for the regions of extreme sparsity ($< 5\%$ or $> 95\%$), where sdarray achieves a smaller representation size.

Finally, in the smallrank scheme, we adaptively switch between small and stat without the select support data structure. This representation achieves exceptionally good compression while providing a very good query time for access and rank queries.

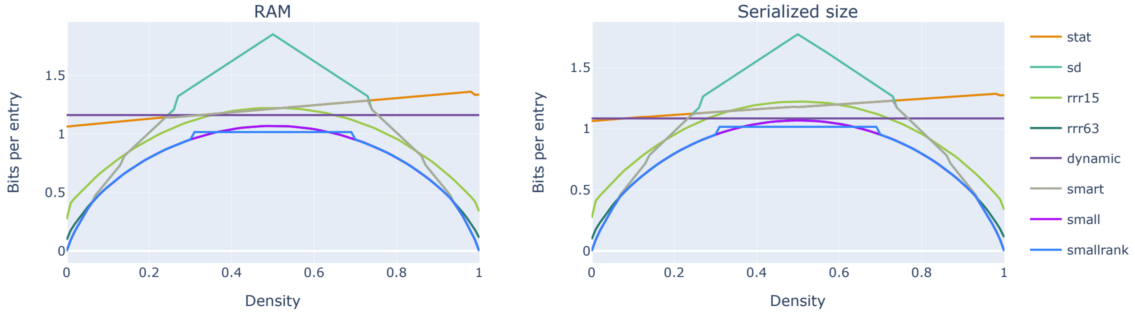

Supplementary Figure S-1: Size of different bitmap representations (in bits per entry), with set bits uniformly distributed in bitmaps of size 10^9 and different densities. **Left:** RAM required to load the representation. **Right:** disk space used to store the representation.

A.3 Indexing sequences in de Bruijn graphs

By definition, the de Bruijn graph of order k is a pair (V, E), where nodes V are a set of k-mers, and directed edges E are all its node pairs $(s_1, s_2) \in V^2$, where the longest nontrivial suffix of the source k-mer s_1 matches the longest nontrivial prefix of the target k-mer s_2. The edges E can be unambiguously derived from the nodes V. Thus, to represent a de Bruijn graph, it is sufficient to store only its k-mers.

To index the input sequence data, MetaGraph builds a de Bruijn graph from the input sequences and uses it for two purposes. First, the de Bruijn graph serves as an efficient k-mer dictionary and allows mapping the k-mers onto positive integer indices. Second, the graph topology allows setting

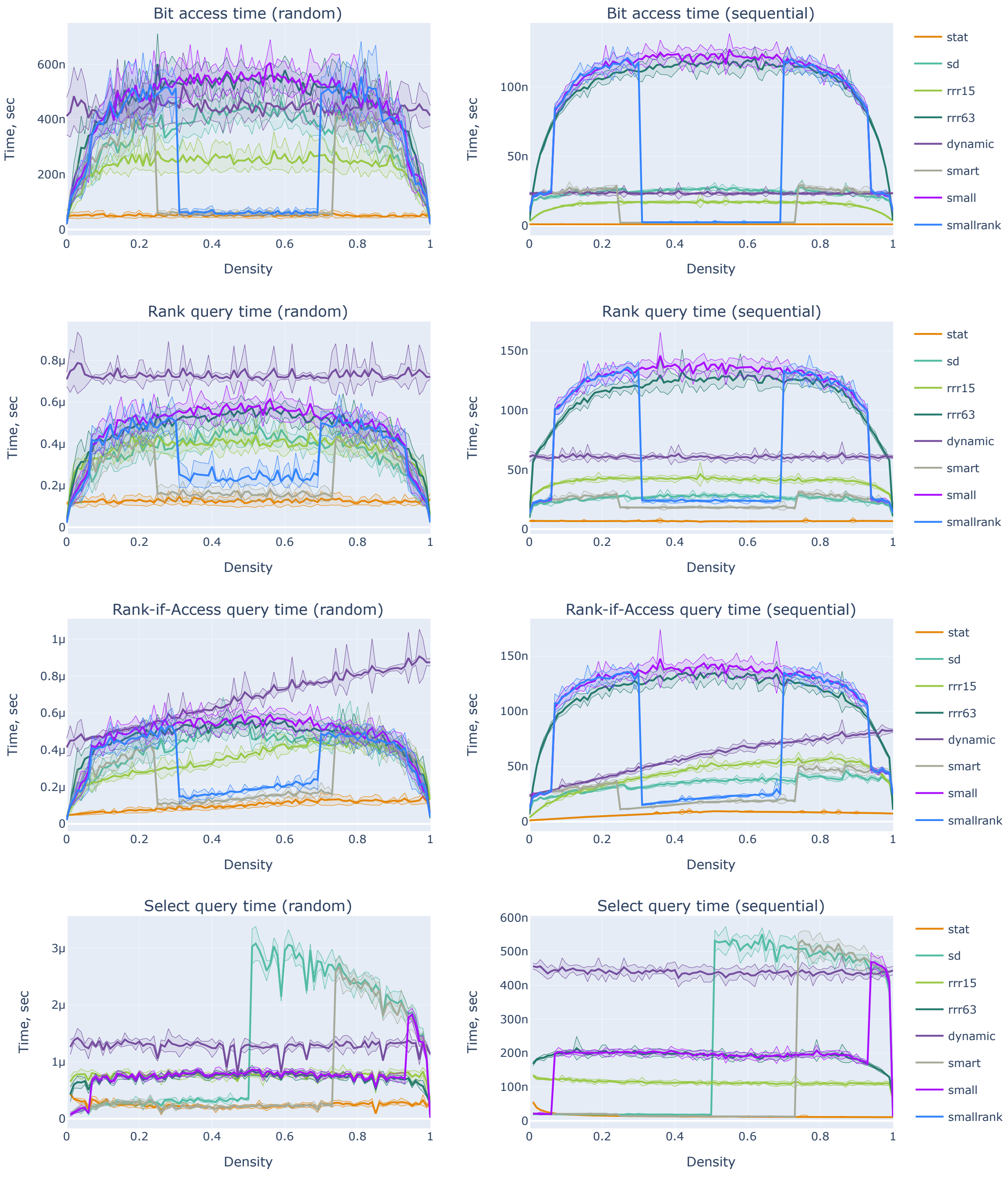

Supplementary Figure S-2: Average query time for different operations (bit access, rank, rank-if-access, and select queries) for different bitmap representations, with set bits uniformly distributed in bitmaps of size 10^9 and different densities. **Left:** when queried at random positions. **Right:** average time per query when queried at consecutive positions. The rank-if-access operation combines access and rank and is defined at position i as rank(i) if access(i) = 1 and zero otherwise.

the inverse problem of assembling sequences from the k-mers into which the input sequences have been initially decomposed.

A.3.1 Representing a de Bruijn graph

MetaGraph employs several data structures (summarized in **Supplementary Table S-1**) for storing k-mer sets, which are used as a basis to implement different representations of the *de Bruijn graph* abstraction: i) a hash table (StrHashDBG and HashDBG), ii) an indicator vector (BitmapDBG), a binary vector represented as a succinct bitmap of size $|\Sigma|^k$ indicating which k-mers are present in the set [93], and iii) the BOSS table [94] proposed by **B**owe, **O**nodera, **S**adakane, and **S**hibuya, storing a set of k-mers succinctly (SuccinctDBG). All these data structures support exact membership queries, and they map k-mers to positive indexes from 1 to n, where n is the number of k-mers in the represented set (or zero if the queried k-mer does not belong to the set). While HashDBG is mostly used internally (e.g., for batched sequence search, see Methods Section "Batched sequence search"), SuccinctDBG and BitmapDBG exhibit the best compression performance for practical use, depending on the value of k. In the next sections below, we describe these data structures in detail.

Supplementary Table S-1: List of graph representations provided in MetaGraph.

**Graph repr.**	$k_{\max}$	$k_{\max}$ **for DNA**	**Bits per k-mer**
StrHashDBG	unlimited	unlimited	$8k + O(1)$
HashDBG	$\lfloor \frac{256}{\lceil \log_2 \|\Sigma\| \rceil} \rfloor$	128	$\max(64, 2^{\lceil \log_2 (k \lceil \log_2 \|\Sigma\| \rceil) \rceil})$
BitmapDBG	$\lfloor \frac{63}{\lceil \log_2 \|\Sigma\| \rceil} \rfloor$	31	$2 + \lceil \log_2 \frac{\|\Sigma\|^k}{n} \rceil + o(1)$ [93]
SuccinctDBG	$\lfloor \frac{256}{\lceil \log_2 \|\Sigma\| + 1 \rceil} \rfloor$	85	$2 + \log_2 \|\Sigma\| + o(1)$ [94]

Hash table-based de Bruijn graph representations

As a basic de Bruijn graph representation, we use a general-purpose hash table to map k-mers packed into 64, 128, or 256-bit integers to their positive integer identifiers (all k-mers are numbered from 1 to n). Although this representation is very space-consuming, it enables dynamic insertion and deletion operations and is very useful for algorithm prototyping. In addition, with k-mers stored as strings (with $8k + O(1)$ bits per k-mer), this representation automatically supports k-mers of arbitrary length, which may be important in some applications. As the underlying hash table supports insertions, these representations can naturally be used as dynamic de Bruijn graph representations.

Representing a k-mer dictionary with an indicator bitmap

Another de Bruijn graph representation we implemented is BitmapDBG, which encodes the presence of k-mers in an indicator vector. Being of size $|\Sigma|^k$ and having set bits in those and only those positions that correspond to the present k-mers, this indicator vector represents a k-mer dictionary and, hence, its respective de Bruijn graph. This scheme was originally proposed in [93]. We store the indicator vector in an sdarray compressed representation [91], which takes for a vector of size N with n set bits $n(2 + \lceil \log_2 \frac{N}{n} \rceil) + o(n)$ bits of space (or, equivalently, $2 + \lceil \log_2 \frac{|\Sigma|^k}{n} \rceil + o(1)$ bits per k-mer, where n is the total number of k-mers in the dictionary) and performs rank operations in $O(\log \frac{N}{n})$ time, as implemented in the *sdsl-lite* library [91].

A.3.2 Succinct k-mer dictionaries represented as a BOSS table

The most scalable and in many ways most versatile de Bruijn graph representation available in MetaGraph is SuccinctDBG. It is based on the succinct self-index proposed by **B**owe, **O**nodera, **S**adakane and **S**hibuya [94] and referred to in the literature as the *BOSS table*. While **Supplementary Table S-1** only shows the theoretical size of the BOSS table (SuccinctDBG) according to estimations in [94], in practice, we developed three different versions of this representation: SuccinctDBG (static) (for fast queries), SuccinctDBG (dynamic) (supporting k-mer insertions), and SuccinctDBG (small) (a good space vs. time trade-off, in practice typically taking just around 2 or 3 bits per k-mer).

While SuccinctDBG often achieves the best compression of the k-mer dictionary, which becomes a game-changer when indexing data at Petabase scale, this scalability comes at the cost of slower k-mer membership queries to retrieve the respective node indexes in the graph, requiring up to k internal traversal steps [94] for each k-mer query. We alleviate this issue by augmenting the BOSS table with an auxiliary table mapping the k-mer suffixes (usually of length 12) to their respective ranges of rows in the BOSS table (see more details in Section A.3.2). With this optimization and the careful implementation in general, SuccinctDBG achieves a performance, which is sufficient to make the overall query times competitive to other methods while keeping the memory footprint at least an order of magnitude lower (see **Figure 2 a,b**).

In the sections below, we describe this succinct representation in detail, as well as the algorithms for its construction and performing basic operations such as graph traversal and k-mer query.

The BOSS representation

The BOSS table [94] consists of three vectors W, F, and L (defined below) and represents a de Bruijn graph where each node has at least one incoming and at least one outgoing edge (later we show how to loosen this requirement). To satisfy this condition, we extend the alphabet with a special sentinel character $\texttt{\$}$ and add k extra sentinel characters to the beginning and end of every input sequence: $\hat{s} := \texttt{\$}^k\, s\, \texttt{\$}^k \in \hat{\Sigma}^* \;\forall s \in S$. Here $\hat{\Sigma} := \Sigma \cup \{\texttt{\$}\}$ denotes the extended alphabet (i.e., $\hat{\Sigma} = \{\texttt{\$}, \texttt{A}, \texttt{C}, \texttt{G}, \texttt{T}\}$ for DNA) and $\hat{\Sigma}^*$ denotes the set of all finite sequences over this alphabet. As each k-mer, according to this condition, has at least one child and one parent adjacent to it in the de Bruijn graph, we will call a k-mer *first incoming* (*last outgoing*) if there are no colexicographically smaller (larger) k-mers with the same suffix (prefix) of length $k-1$. (Colexicographic order is obtained by reversing all strings/k-mers, applying lexicographic order, and reversing them again.) The BOSS table requires all k-mers to be sorted in colexicographic order starting from the penultimate character, with ties broken by the last characters. That is, a k-mer $s'_1 \cdots s'_k$ precedes another k-mer $s''_1 \cdots s''_k$ if k-mer $s'_k\, s'_1 \cdots s'_{k-1}$ is colexicographically less than k-mer $s''_k\, s''_1 \cdots s''_{k-1}$:

$$s'_k\, s'_1 \cdots s'_{k-1} \prec^{\text{colex}} s''_k\, s''_1 \cdots s''_{k-1}, \tag{A.3}$$

which equivalently corresponds to $s'_{k-1} \cdots s'_1\, s'_k$ being lexicographically smaller than $s''_{k-1} \cdots s''_1\, s''_k$. Given a sorted list of k-mers $(e^{(1)}, \ldots, e^{(n)})$, where $e^{(j)} = e^{(j)}_1 \cdots e^{(j)}_k \equiv e^{(j)}_{1\ldots k-1} e^{(j)}_k \in \hat{\Sigma}^k$, the BOSS

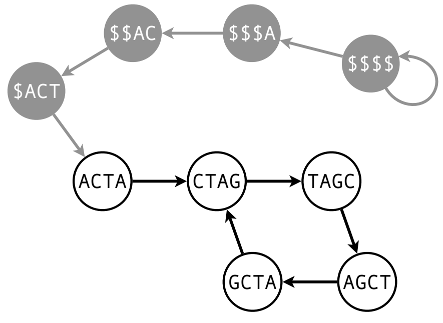

Supplementary Figure S-3: De Bruijn graph constructed for all k-mers extracted from sequence `ACTAGCTAGCTAGC` where $k = 4$.

#	k-mer	L	F	W
1	`$$$$`	0	`$`	`$`
2	`$$$A`	1	`$`	`A`
3	`$$AC`	1	`A`	`C`
4	`CTAG`	1	`A`	`G`
5	`$ACT`	1	`C`	`T`
6	`AGCT`	1	`C`	`T`
7	`TAGC`	1	`G`	`C`
8	`ACTA`	1	`T`	`A`
9	`GCTA`	1	`T`	`A-`

Supplementary Table S-2: The BOSS table representation of all k-mers extracted from sequence `ACTAGCTAGCTAGC` where $k = 4$.

table encodes them in arrays W', L', L, and F defined as follows:

$$\begin{aligned}
W' &= (e_k^{(1)}, \dots, e_k^{(n)}), \\
L' &= (L'_1, \dots, L'_n), \quad L'_j = \begin{cases} 1 & \text{if } e_{2\dots k}^{(t)} \prec^{\text{colex}} e_{2\dots k}^{(j)} \ \forall t < j, \\ 0 & \text{otherwise,} \end{cases} \quad j = 1, \dots, n, \\
L &= (L_1, \dots, L_n), \quad L_j = \begin{cases} 1 & \text{if } e_{1\dots k-1}^{(t)} \succ^{\text{colex}} e_{1\dots k-1}^{(j)} \ \forall t > j, \\ 0 & \text{otherwise,} \end{cases} \quad j = 1, \dots, n, \\
F &= (e_{k-1}^{(1)}, \dots, e_{k-1}^{(n)}).
\end{aligned} \tag{A.4}$$

That is, $W' \in \hat{\Sigma}^n$ is the array of the last characters of the indexed k-mers, $L' \in \{0,1\}^n$ indicates *first incoming* k-mers (see definition above) in the de Bruijn graph, $L \in \{0,1\}^n$ similarly indicates *last outgoing* k-mers, and $F \in \hat{\Sigma}^n$ is the array of the penultimate characters of the k-mers. Finally, the W' and L' arrays are combined into one vector W as follows:

$$\begin{aligned}
W &= ((W'_1, L'_1), \dots, (W'_n, L'_n)) \\
&= ((e_k^{(1)}, L'_1), \dots, (e_k^{(n)}, L'_n)) \in (\hat{\Sigma} \times \{0,1\})^n,
\end{aligned} \tag{A.5}$$

and the vectors F, L, and W comprise the BOSS representation of the de Bruijn graph corresponding to the list of indexed k-mers $(e^{(1)}, \dots, e^{(n)})$. In practice, however, we extend the alphabet and encode every pair $(c, 0)$, where $c \in \hat{\Sigma}$, by adding a symbol '-' to it (e.g., `G-`), while every pair $(c, 1)$ is encoded as the same character $c \in \hat{\Sigma}$. As an example, the BOSS table for a de Bruijn graph from **Supplementary Figure S-3** is shown in **Supplementary Table S-2**. A demonstration tool for constructing BOSS tables for arbitrary nucleotide sequences is available at `https://metagraph.ethz.ch/dbg_visualizer`. Note that due to the specific ordering of k-mers in the BOSS table, vector F can be fully represented by $|\Sigma|$ integers, offsets indicating for every $c \in \Sigma$ how many characters lexicographically less than c occur in vector F, that is, $F_{\text{offset}}(c) := |\{i \mid F_i < c\}|$.

k-mer traversals

In this paragraph, we will show how the BOSS table (i.e., vectors F, L, and W) allows traversing the represented de Bruijn graph along the edges forward and backward.

Forward traversal. The problem of *forward traversal* is set as follows. Given an index j, $1 \leq j \leq n$, of k-mer $e^{(j)} \in V$ in the BOSS table (where $V \subset \hat{\Sigma}^k$ denotes the set of represented k-mers) and a character $c \in \Sigma$, find index j' associated with its successor (child node) $e' = e_{2,\dots,k}\,c$ or 0 if such k-mer does not belong to the graph:

$$\mathrm{fwd}(j, c) := \begin{cases} j' : & e^{(j')} = e^{(j)}_{2,\dots,k}\,c \quad \text{if } e^{(j)}_{2,\dots,k}\,c \in V, \\ 0 & \text{otherwise.} \end{cases} \tag{A.6}$$

To execute this operation and find index j', we exploit the specific ordering of the k-mers in the BOSS table. One can always transition to the last outgoing k-mer by computing the index of the last outgoing k-mer for $e^{(j)}$

$$\mathrm{fwd}(j) = \mathrm{select}^L_1\left(\mathrm{rank}^L_1\left(F_{\mathrm{offset}}(e^{(j)}_k)\right) + \mathrm{rank}^W_{e^{(j)}_k}(j)\right), \tag{A.7}$$

and checking the array W for characters c and $c-$ at positions $\mathrm{select}^L_1(\mathrm{rank}^L_1(\mathrm{fwd}(j) - 1)) + 1, \dots, \mathrm{fwd}(j)$ corresponding to all the k-mers outgoing from $e^{(j)}$ to identify whether one of them ends with character of interest c (see **Supplementary Table S-2**, e.g., $\mathrm{fwd}(6) = 9$ and $\mathrm{fwd}(6, \mathtt{A}) = 9$). Here $\mathrm{select}^L_1(x)$ returns the position of the x-th set bit in array L, $\mathrm{rank}^W_c(j)$ returns the number of times character c occurs in array W up to position j (inclusive), and $F_{\mathrm{offset}}(c)$ returns the number of all characters in array F lexicographically smaller than c. In practice, we precompute $\mathrm{rank}^L_1(F_{\mathrm{offset}}(c))$ for all $c \in \hat{\Sigma}$ and store these $|\hat{\Sigma}|$ integer values.

Backward traversal. We define the *blind backward traversal* as follows. Given a k-mer $e^{(j)} \in V \subset \hat{\Sigma}^k$ and associated with it index j in the BOSS table, find the first incoming k-mer for it and return its associated index

$$\mathrm{bwd}(j) = j', \quad \text{where } e^{(j')}_{2,\dots,k} = e^{(j)}_{1,\dots,k-1} \text{ and } W_{j'} \in \hat{\Sigma}. \tag{A.8}$$

Note that the last condition $W_{j'} \in \hat{\Sigma}$ is equivalent to $L'_{j'} = 1$ and implies that j' is the first k-mer incoming to k-mer $e^{(j)}$. The existence of the solution follows from the existence of at least one incoming edge for each k-mer of the de Bruijn graph encoded in the BOSS table. One can see that this can be computed on arrays L, F, and W of the BOSS table as follows:

$$\mathrm{bwd}(j) = \mathrm{select}^W_{e^{(j)}_{k-1}}\left(\mathrm{rank}^L_1(j-1) + 1 - \mathrm{rank}^L_1\left(F_{\mathrm{offset}}(e^{(j)}_{k-1})\right)\right). \tag{A.9}$$

Indeed, it is easy to see that $\mathrm{rank}^L_1(\mathrm{fwd}(\mathrm{bwd}(j)) - 1) = \mathrm{rank}^L_1(\mathrm{select}^L_1(\mathrm{rank}^L_1(j-1) + 1) - 1) = \mathrm{rank}^L_1(j-1)$, as $\mathrm{rank}^A_1(\mathrm{select}^A_1(\mathrm{rank}^A_1(x) + 1) - 1) = \mathrm{rank}^A_1(x)\ \forall x, A$. That is, $\mathrm{bwd}(j)$ returns a node, which is mapped by $\mathrm{fwd}(\cdot)$ back to j or to another k-mer that shares with j its source (or, equivalently, has the same prefix of length $k-1$).

Decoding k-mers

Note that given an index j of a k-mer in the BOSS table, one can immediately identify the last character of the k-mer from vector W of the BOSS table (see **Supplementary Table S-2**). Then, with the blind backward traversal, one can transition to an adjacent incoming k-mer and repeat the same operation to extract the next character of the original k-mer. Repeating this procedure k times reconstructs the original k-mer by decoding all its characters.

k-mer lookups

In Section A.3.2, we described how given a node index, one can traverse the de Bruijn graph to transition to one of its adjacent nodes. Here, we will describe the algorithm for k-mer lookup. More precisely, given a k-mer $c_1 \cdots c_k$, find its index in the BOSS table or return 0 if such k-mer is not encoded in the BOSS table. We start with the range of k-mers in the BOSS table that have character c_1 in vector F. That is, all k-mers with pattern $* \cdots * c_1 *$ (recall that vector F in the BOSS table encodes penultimate characters of the k-mers stored in it while vector W encodes the last characters of the k-mers). Thanks to the specific order of the k-mers in the BOSS table, this range is continuous, and thus, we denote it as $[\mathrm{first}_1, \mathrm{last}_1]$ (e.g., $[\mathrm{first}_1, \mathrm{last}_1] = [5, 6]$ for character `C` in the example from **Supplementary Table S-2**). Then, we tighten this range by moving pointer first_1 to the next occurrence of c_2 in vector W, and pointer last_1 to the preceding occurrence of c_2 in W. Suppose, our range after this step is $[\mathrm{first}'_1, \mathrm{last}'_1] \subseteq [\mathrm{first}_1, \mathrm{last}_1]$. Now we make the forward transition with operation fwd and transform the range to $[\mathrm{first}_2, \mathrm{last}_2] := [\mathrm{select}_1^L(\mathrm{rank}_1^L(\mathrm{fwd}(\mathrm{first}'_1) - 1)) + 1, \mathrm{fwd}(\mathrm{last}'_1)]$, where fwd is defined by formula (A.7). As a result, the new range $[\mathrm{first}_2, \mathrm{last}_2]$ corresponds to all the k-mers of pattern $* \cdots * c_1 c_2 *$ encoded in the BOSS table. After repeating this procedure $k - 3$ more times, we get a range corresponding to k-mers $c_1 c_2 \cdots c_{k-1} *$ and complete the operation by checking W for c_k and c_k- within that range to locate the k-mer of interest $c_1 c_2 \cdots c_k$. If during the execution of the algorithm a range $[\mathrm{first}'_i, \mathrm{last}'_i]$ becomes invalid (i.e., $\mathrm{first}'_i > \mathrm{last}'_i$), this implies that the k-mer being searched does not belong to the graph, and hence, we return 0.

Also note that by interrupting this algorithm early, after $k' < k$ iterations, we effectively locate all the k-mers with a given suffix (more precisely, the k-mers of pattern $* \cdots * c_1 \cdots c_{k'} *$). We call this operation the *sub-k-mer matching*. Sub-k-mer matching is essential for inexact sequence search algorithms described in the sections below. It could also be efficiently implemented with the BitmapDBG representation of de Bruijn graphs, but it would be impossible to perform with hash table-based representations without an exhaustive search.

Indexing k-mer ranges by suffix

At the t-th iteration of the k-mer lookup algorithm described above, the current range of nodes corresponds to the k-mers in the BOSS table of pattern $* \cdots * c_1 \cdots c_t *$. Thus, if all these ranges were precomputed for all k-mer suffixes $c_1 \cdots c_t \in \Sigma^t$, one could immediately get the respective range for any given k-mer suffix and thereby skip the first t iterations of the lookup algorithm. At worst, this would reduce the query time by a factor of $\frac{k}{k-t}$. In the best case, however, this single lookup in the vector of precomputed k-mer ranges would be enough to find that there are no k-mers in the BOSS table with the given suffix. Hence, it would reduce the entire k-mer lookup algorithm to this single lookup in the vector of precomputed k-mer ranges.

We implemented this idea in MetaGraph and found it to be very effective for speeding up queries on the BOSS table. We call this vector of precomputed ranges an *index of suffix ranges* and represent it with the compressed `sdarray` [91] bitmap. More precisely, in this representation, we encode a sparse bitmap $SR \in \{0, 1\}^{1+n+2|\Sigma|^t}$, where n is the size of the BOSS table (the number of k-mers), with set bits at positions indicating the borders of the suffix ranges (hence, of size $1 + n + 2|\Sigma|^t$ with $2|\Sigma|^t$ set bits). Namely, the range of k-mers with the p-th suffix (out of all $|\Sigma|^t$ possible suffixes) in the BOSS table can be computed as $\left[\mathrm{select}_1^{SR}(2p + 1) - 2p,\ \mathrm{select}_1^{SR}(2p + 2) - 2p - 1\right)$. This representation requires only about $2|\Sigma|^t \left(2 + \log_2 \frac{1+n+2|\Sigma|^t}{2|\Sigma|^t}\right)$ bits, hence, approximately $4 + 2\log_2 \frac{n}{2|\Sigma|^t}$ bits per integer in the table of ranges when $|\Sigma|^t \ll n$. For instance, with $n = 100 \cdot 10^9$, $t = 12$, and $|\Sigma| = 4$, this makes up about $4 + 2\log_2 \frac{100 \cdot 10^9}{2 \cdot 4^{12}} \approx 27$ bits per range instead of the 128

bits, which we would need if we stored each range with a pair of 64-bit integers. In addition to the space savings, this representation allows querying the ranges in $O(1)$ time (the complexity of the $\text{select}_1(\cdot)$ operation) as well as performing inverse queries (find a suffix of a k-mer given its index in the BOSS table) in $O(\log |\Sigma|^t)$ time. Even though the complexity of this operation is asymptotically the same as t backward traversal steps on the BOSS table, in practice, the constant is much lower, which makes the computation of k-mer suffixes this way significantly faster than when directly traversing the BOSS table.

Dummy k-mers in the BOSS table

Since we only make traversal steps from real k-mers encoded in the BOSS table and never traverse forward *dummy sink* k-mers (those ending with the sentinel character $\$ \notin \Sigma$), the requirement of the existence of at least one outgoing edge in the represented de Bruijn graph has to be applied only to real k-mers, that is, those without sentinel characters `$`. Thus, for each original real k-mer e without adjacent outgoing k-mers, we only need to add a single dummy k-mer $e_{2,\ldots,k}\$$ to the BOSS table (see **Supplementary Figure S-4**).

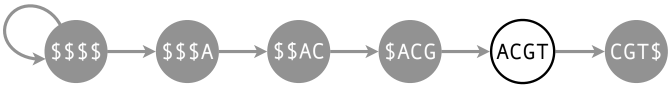

Supplementary Figure S-4: De Bruijn graph for k-mer `ACGT` with all required non-redundant dummy k-mers.

Redundant dummy k-mers and their removal. Some operations over de Bruijn graphs (e.g., merging two graphs) may lead to the existence of so-called *redundant dummy k-mers* in the graph, that is, dummy k-mers (with at least one sentinel character) that can be removed from the BOSS table without breaking the properties required for graph traversals. For example, a sink dummy k-mer $c_1 \cdots c_{k-1}\$$ is redundant if there is another k-mer $c_1 \cdots c_{k-1}c_k$ that belongs to the graph, where $\$ \neq c_k \in \Sigma$. Redundant dummy source k-mers are defined analogously. In MetaGraph, we implemented special procedures to identify and erase these redundant dummy k-mers from the BOSS table in linear time.

Internal representations of the BOSS table

For different use cases that arise in practice, we developed three different implementations of the BOSS table (L, F, W), which we call *graph states*. These different graph states tune the whole graph structure to requirements implied by a particular problem setting and available computational resources. We also present procedures for internal conversion between all the implemented graph states. Given a graph in one state, it can be easily converted to any other.

Static representation. In the default static representation, which provides a very good query performance while only taking about $2 + \log_2 |\Sigma|$ bits per k-mer, vector L is stored as an uncompressed packed bitmap with the default rank and select support data structures from the *sdsl-lite* library [91] ensuring constant time *rank*, *select*, and *access* operations (see the `stat` bitmap representation in Section A.2), vector W is encoded using the Huffman wavelet tree implemented in the *sdsl-lite* library [91]. Vector F, as always, is simply represented as an array of $|\Sigma|$ offsets.

Small representation. For the smallest memory footprint, we encode the internal bitmaps of the Huffman wavelet tree W in RRR vectors implemented in the *sdsl-lite* library [91], which ensures a nearly optimal compression. Similarly, vector L is encoded in a hybrid small representation (see Section A.2.2) switching between RRR and sdarray [91] vectors, depending on the sparsity of vector L to ensure the best compression. Vector F is encoded by the offset array as described above.

Dynamic representation. To support dynamic operations on the BOSS table, we use dynamic bit vector and string representations based on the cache-efficient B-trees implemented in library DYNAMIC [92] to represent vectors W and L with the support for dynamic insertion and deletion operations. Vector F is encoded by the offset array as described above and naturally supports constant-time dynamic operations.

In our workflows, we usually first construct a de Bruijn graph in the SuccinctDBG (static) representation and then convert it to SuccinctDBG (small) once this graph has been annotated and the entire MetaGraph index is constructed and ready to serve queries.

A.3.3 Graph construction

While the StrHashDBG and HashDBG graph representations are constructed directly by inserting k-mers into a hash table, the more advanced representations BitmapDBG and SuccinctDBG require a sorted list of k-mers for their construction. Thus, the construction from a set of input sequences proceeds in two steps: 1) k-mer extraction, sorting, and de-duplication; 2) construction of the representation (an indicator bitmap or the BOSS table). In the first stage, each k-mer e extracted from the input sequences, depending on the alphabet size and the k-mer length k, is represented as a 64-, 128-, or 256-bit integer, where each character is encoded with exactly $b = \lceil \log_2 |\Sigma| \rceil$ bits ($b = \lceil \log_2 |\hat{\Sigma}| \rceil$ when constructing the BOSS table). The layout of the characters in k-mers is defined accordingly to induce the relative order of the k-mers expected in the target data structure. In the case of the BOSS table, in particular, this is the colexicographic order described in Section A.3.2. Once all k-mers have been de-duplicated and sorted, we construct the final representation, that is, a compressed indicator bitmap for BitmapDBG or vectors L, W, and F of the BOSS table for SuccinctDBG.

Distributed construction

For large data sets where the full set of encoded k-mers may not fit in the amount of RAM available, we employ a distributed construction approach. Each process is assigned a suffix of a fixed length ℓ and only k-mers with that suffix are extracted and sorted by that process. The resulting $C_i = (W_i, L_i, F_i)$ tuples from these processes are referred to as *graph chunks*, from which the full graph representation can be constructed through index-wise concatenation. More precisely, given graph chunks $C_1, \ldots, C_{|\hat{\Sigma}|^\ell}$ sorted by their respective suffixes, the final graph representation is

$$W := W_1 \cdots W_{|\hat{\Sigma}|^\ell}, \quad L := L_1 \cdots L_{|\hat{\Sigma}|^\ell}, \quad F := \sum_{i=1}^{|\hat{\Sigma}|^\ell} F_i. \tag{A.10}$$

A.4 Representing binary matrices in MetaGraph

A.4.1 Column-major matrix representations

The default representation of graph annotations in MetaGraph is *ColumnCompressed*, which independently stores columns as compressed bit vectors. More precisely, in the hybrid bit vector representation smart (described in Section A.2.2). Being highly compressed for sparse columns, this representation provides easy access to individual columns, which is helpful for filtering (e.g., by sample IDs) and selecting label-induced subgraphs. Furthermore, the *ColumnCompressed* representation provides efficient *access* queries, which makes it an excellent choice for querying individual columns. At the same time, the set bits of a column stored in the smart representation can be iterated exceptionally quickly, and thus, the entire matrix can be easily transformed to any other format, including row-major formats, for which the matrix is effectively transposed by blocks.

For higher compression performance and faster row queries, we employ the Multiary Binary Relation Wavelet Tree (*Multi-BRWT*) representation scheme [95] with the index columns stored in the compressed smallrank representation (see Section A.2.2), which provides excellent compression while enabling fast rank queries. The *Multi-BRWT* representation scheme typically achieves the best compression in real applications, especially where the columns of the annotation matrix are highly correlated, such as those constructed from sequencing samples corresponding to related organisms.

Generally, when performing row queries on an annotation matrix represented with a column-major scheme, we aggregate the query operations and perform them in batches, which improves the cache locality and significantly improves the query performance.

A.4.2 Row-major matrix representations

For fast queries on rows (e.g., for sequence search queries), we provide a number of row-major matrix representations. For the fastest queries of rows, we developed a compressed row-major sparse matrix representation *RowCompressed*, which is employed in query graphs (**Extended Data Figure 1 e**) and additionally supports dynamic operations. It stores the matrix as a vector of vectors, where each row is represented as a `folly::SmallVector<uint32_t>` from the Facebook Open-source Library[1], storing the column indexes of its set bits, which has the same interface as `std::vector<uint32_t>` but significantly lower memory overhead.

The next compression technique, *RowFlat*, was originally employed in VARI [96]. It concatenates all rows into a single sparse bitmap of size mn, where n is the number of rows and m is the number of columns of the annotation matrix, and stores this bitmap in a compressed sdarray [91] representation.

Then, *RowSparse* writes the column indexes of all set bits into an integer array compressed with the Elias delta coding (implemented in the `sdsl::vlc_vector<>` data structure from the *sdsl-lite* library [91]). Additionally, a bitmap of size $n + d$, where d is the number of set bits in the matrix, is stored in the small representation (described in Section A.2.2) to encode the offsets pointing to where each row starts in that integer array.

In *BinRel-WT* [97], a similar approach is used with the difference that the integer array is represented as a wavelet tree, which improves the performance of column queries.

Next, *Rainbowfish* [98] uses the fact that rows of the annotation matrix are often highly duplicated (due to multiple nodes in the graph having the same annotations). To take advantage of that, it builds a dictionary of distinct rows and stores a mapping from original row indexes to their corresponding indexes of distinct rows in the dictionary. Additionally, the more frequent rows

[1] `https://github.com/facebook/folly`

are assigned smaller indexes in the dictionary, which makes this mapping more compressible with universal codes. Interestingly, this technique can be generalized and used in combination with any matrix representation scheme. We describe this generalization below.

A.4.3 The Rainbow matrix decomposition technique

It is easy to see that the row de-duplication technique used in *Rainbowfish* [98] can generally be used with any compression scheme used to represent the matrix of distinct rows. Moreover, this matrix does not have to be represented in a row-major order but any column-major representation would be applicable as well. We call this technique the *Rainbow* decomposition, and thus, we call the representations employing it the *Rainbow-** representation schemes. For example, the *Rainbow-BRWT* representation de-duplicates the rows with the Rainbow technique and stores the distinct rows in a matrix represented with the compressed *Multi-BRWT* scheme. Note that in this terminology, *Rainbow-RowFlat* refers to the original *Rainbowfish* [98] representation.

A.5 Estimating the cost of comprehensive nucleotide search engine

In this section, we will estimate the cost of constructing and hosting MetaGraph indexes, as well as the cost of serving user queries for sequence search. First, we will make general calculations and then make calculations specifically for hosting the whole SRA.

A.5.1 General estimation methodology and assumptions for MetaGraph

Suppose we would like to index with MetaGraph a certain large data set (such as the SRA) of size D, measured in base pairs. We partition the whole data set into n parts and index them independently. As a result, we get n MetaGraph indexes of size $I_1, \dots, I_n$, which we would host on n machines of a certain type to execute user queries.

Next, we will make the following assumptions.

1. All indexes have the same size $I_1 = \dots = I_n$. Moreover, the total size of the indexes $I := \sum_{i=1}^{n} I_i$ does not depend on the partitioning of the data set and, in particular, on the number of chunks n.

2. The resources required to host an index (memory and local disk space) grow linearly with the index size: $R_M(I) = rI$, $R_L(I) = lI$.

3. The throughput for search and alignment is in inverse dependence with the index size I: $S(I) = s/I$, where $S(I)$ denotes either the throughput of k-mer matching or one of the alignment algorithms in MetaGraph running on an index of size I with a single thread.

As an implication of the first assumption, one can index a part of the whole data set of size D/n and derive from the size I of this index the compression ratio $c = \frac{D}{nI}$. Essentially, the assumption says that the compression ratio does not depend on the partitioning of the data set. In effect, MetaGraph does exploit the redundancy across the indexed samples to enhance the compression. Thus, the estimation of c on a relatively small subset (especially randomly selected) generally leads to an underestimated value of c. In turn, this leads to overestimating the costs of hosting indexes constructed on larger parts of the data set.

The second assumption comes naturally.

The third assumption typically holds true (see the results in **Figure 2b** as an example). Moreover, if, theoretically, the query time grows superlinearly for a certain type of data, the data set can be partitioned into smaller chunks, which would be indexed independently and queried sequentially to keep the growth of the query time linear.

Suppose each hosting machine has N cores, M RAM, L of local disk space, and costs C per time unit. Then, the minimum number of machines required to host the indexes is $n_{\min} := \lceil \max(\frac{rI}{M}, \frac{lI}{L}) \rceil$, where $I = c \cdot D$ is the total size of the indexes (we used the first assumption for this derivation). If all the machines are queried simultaneously, the total throughput of such a system can be up to $s / \frac{I}{n_{\min}} \cdot N = sNn_{\min}/I$. Since the cost of the machines is $n_{\min}C$, the effective query cost is $\frac{n_{\min}C}{sNn_{\min}/I} = \frac{C \cdot I}{sN}$. Note that the effective query cost does not depend on the number of machines $n_{\min}$ used. Indeed, by using more machines, we pay more per time unit, but the task is performed faster. Hence, the total cost is the same.

A.5.2 Hosting scenarios

We will consider the following scenarios of hosting preconstructed MetaGraph indexes. On the one hand, the MetaGraph indexes can be constantly loaded in RAM to be ready to serve user queries.

On the other hand, the MetaGraph indexes can be precomputed and stored in a cloud storage with fast access to allow the user to start an arbitrary number of machines on demand and execute the queries on them. Each machine would download a precomputed index from the common storage and would run the query against it. With a sufficiently large query (in practice 100 Mbp is already sufficient), the time of downloading and initializing a MetaGraph index can be neglected as it is far smaller than the actual time taken to perform the query against this index.

The first scenario assumes we reserved a sufficient number of machines to keep all the indexes loaded in their RAM. This ensures that one can execute queries online. However, it implies high costs for keeping those machines running constantly. Alternatively, we could reserve significantly fewer machines and keep the indexes in their local storage. Thus, to serve a query, each machine would sequentially load the indexes from the local storage into RAM and would perform the search against them. Effectively, this is equivalent to the strategy 'on-demand' described above, except that we would keep a fixed number of machines ready to serve user queries at all times. As a result, we could use cheaper instances (for dedicated use instead of on-demand) provided by the cloud provider, as in the first scenario. As a downside, the query throughput would be significantly lower, and the delay before seeing the query results would be significantly higher than in the first scenario, which would nevertheless not affect the effective query costs, assuming the query is performed in sufficiently large batches (as in the on-demand scenario).

Chapter B

Supplementary Figures

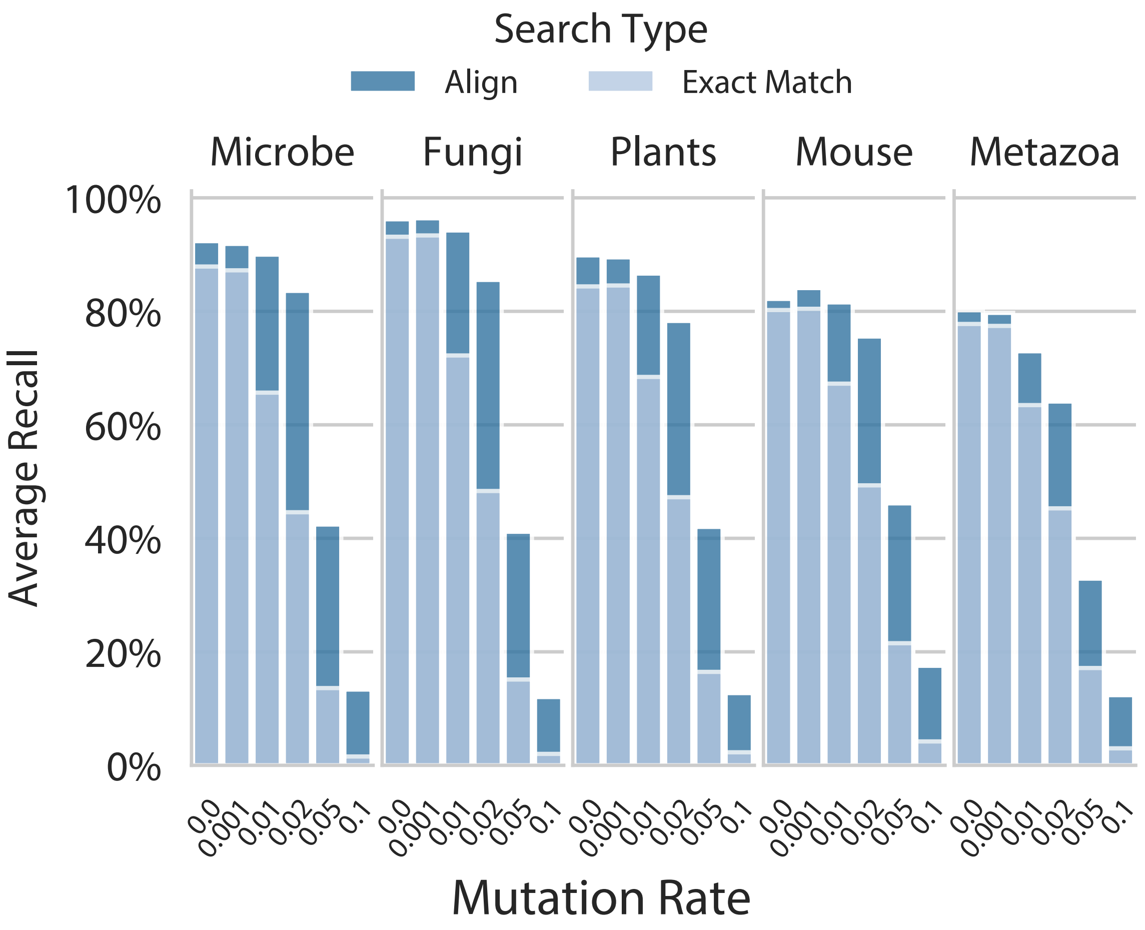

Supplementary Figure S-5: Realignability (average recall) of each graph for increasing error rates in the query (see **Figure 3** for a definition of realignability).

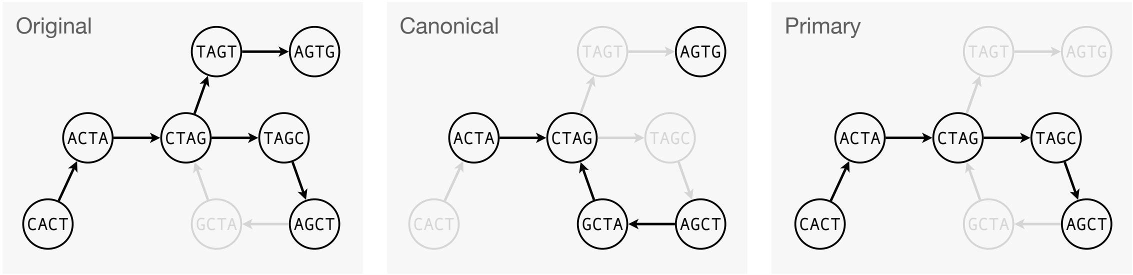

Supplementary Figure S-6: Schematic illustration of canonical and primary graphs. **Left:** A de Bruijn graph of order $k = 4$ constructed from k-mers observed in the input sequences, e.g., `CACTAGCT`, `CTAGTG`. All k-mers (in this case only one k-mer `GCTA`) that did not actually occur in the input but could be present in the sample in reverse complement orientation are dimmed in gray. **Middle:** Canonical graph. All non-canonical k-mers are dimmed in gray and are represented implicitly by the explicitly stored canonical k-mers. For the BOSS table, however, all canonical and non-canonical k-mers are stored in this mode to reduce the number of disconnected components in the graph and thereby minimize the number of extra dummy k-mers. **Right:** Primary graph (i.e., a graph constructed from primary contigs). All non-primary k-mers are dimmed in gray.

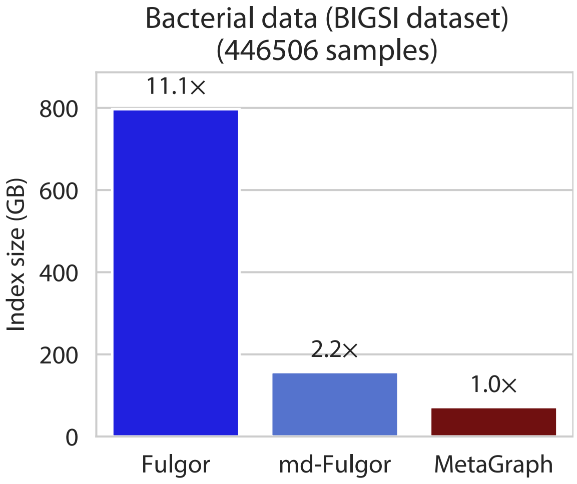

Supplementary Figure S-7: Sizes of indexes constructed from the full SRA-Microbe microbial whole-genome sequencing data set (BIGSI[99]).

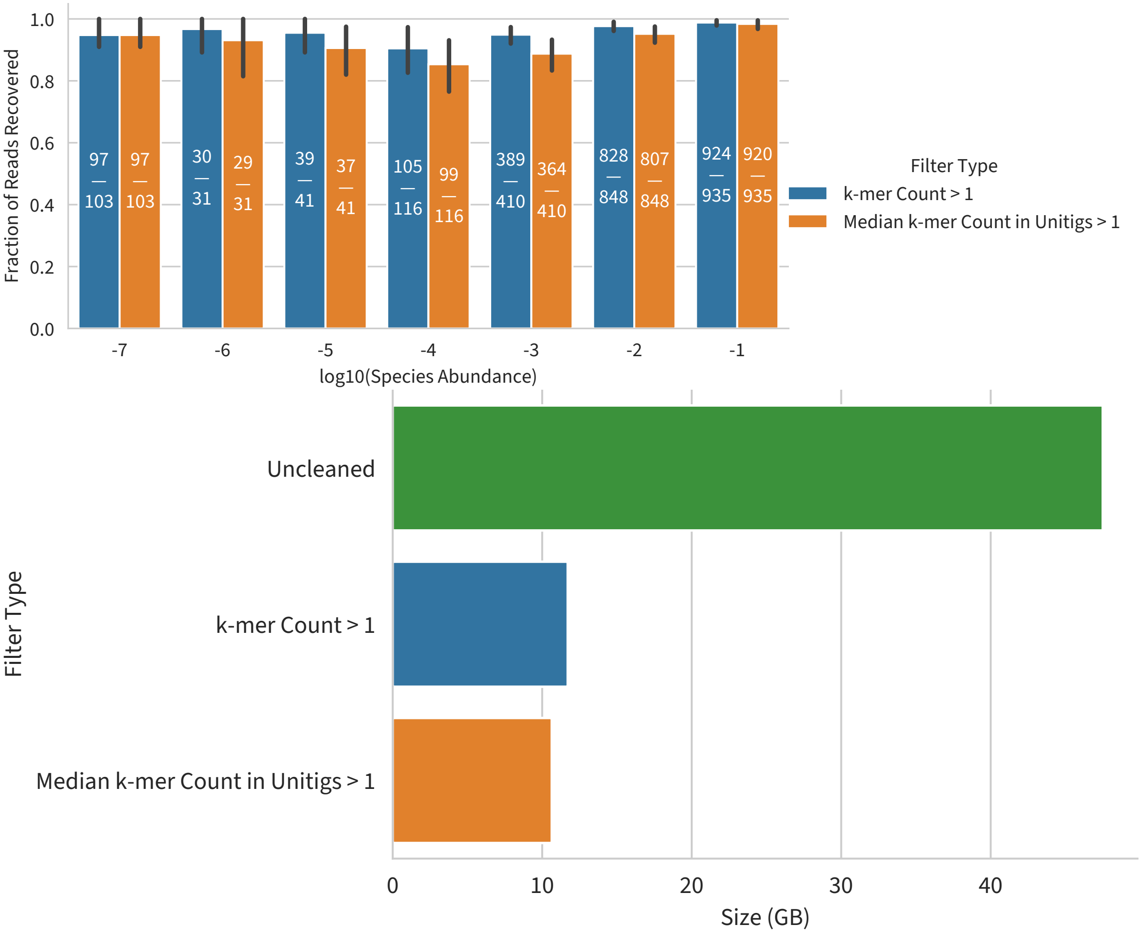

Supplementary Figure S-8: **Top:** Realignability of reads to the SRA-MetaGut graph using a simple singleton filtering strategy (blue) and our unitig-based cleaning strategy (orange) for species at different abundances in their respective original samples. Bars represent the mean recovery percentage from 1000 bootstrap samples of the reads, with error bars representing the 95% confidence intervals of the means. The observed numbers of reads where a species could be determined (correct species recovered / total) are overlaid on the bars. **Bottom:** Sizes of MetaGraph indexes of the SRA-MetaGut data set with different cleaning strategies. All indexes use the SuccinctDBG graph representation and the RowDiff<Multi-BRWT> annotation representation.

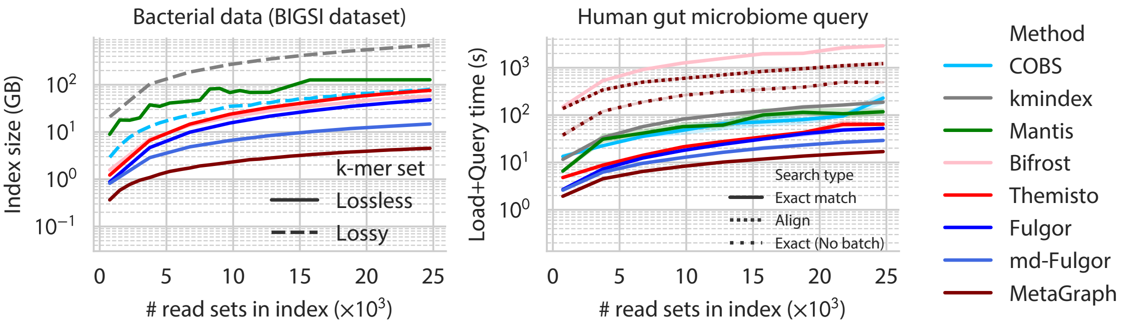

Supplementary Figure S-9: Sizes (**left**) and query times (**right**) of evaluated indexes for growing subsets of microbial whole-genome sequencing samples from SRA-Microbe (from BIGSI[99]). For this read set, the bulk querying approach taken by MetaGraph is on average 28×, and at most 32×, faster than querying each sequence individually.

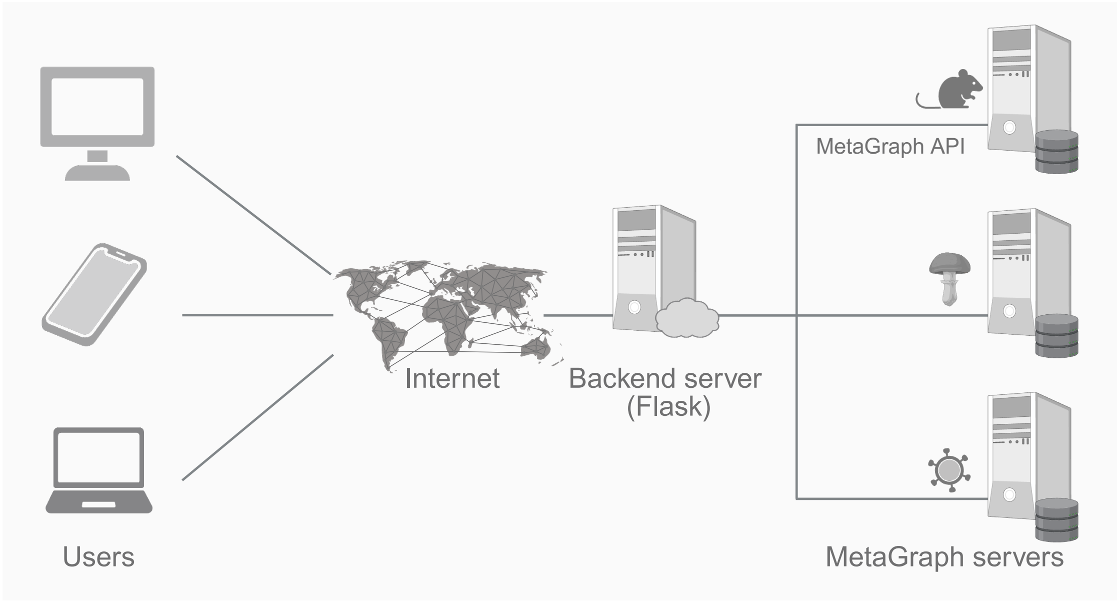

Supplementary Figure S-10: Architecture of MetaGraph Online. – The client-server architecture of MetaGraph Online. The backend server (middle) generates dynamic web pages and transforms user queries to search requests sent to the remote servers hosting MetaGraph indexes. It also provides an API equivalent to that of the MetaGraph server by forwarding requests sent to specific endpoints to their respective MetaGraph servers.

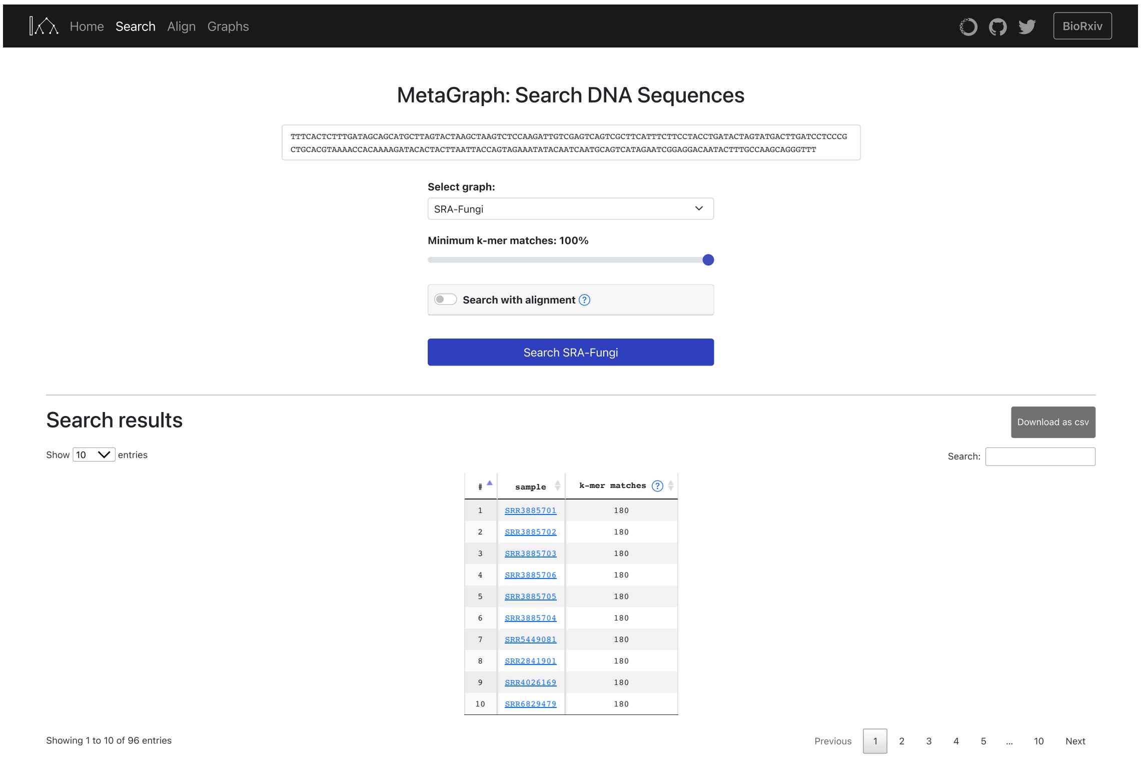

Supplementary Figure S-11: MetaGraph Online. – Web user interface of the MetaGraph Online search engine for sequence search.

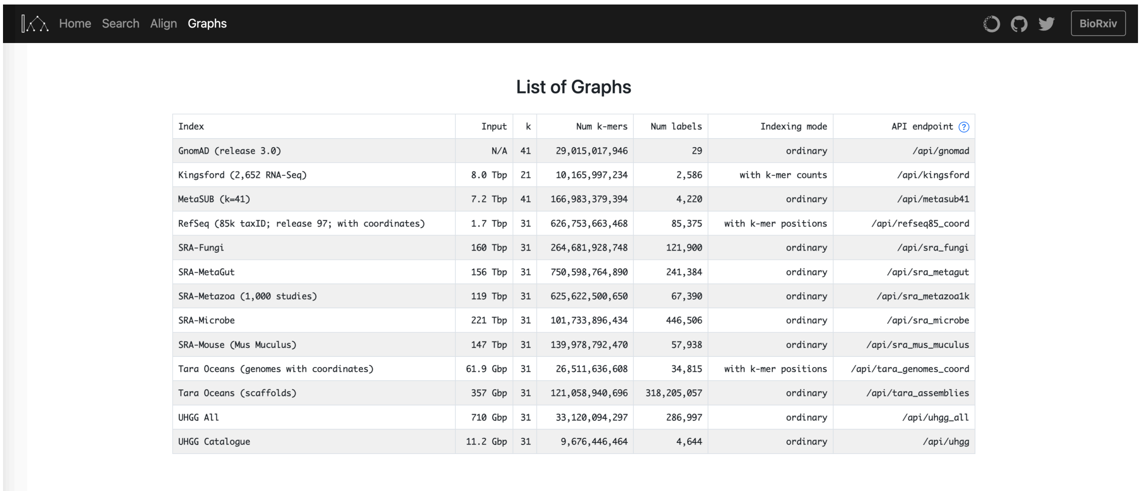

List of Graphs

Index	Input	k	Num k-mers	Num labels	Indexing mode	API endpoint
GnomAD (release 3.0)	N/A	41	29,015,017,946	29	ordinary	/api/gnomad
Kingsford (2,652 RNA-Seq)	8.0 Tbp	21	10,165,997,234	2,586	with k-mer counts	/api/kingsford
MetaSUB (k=41)	7.2 Tbp	41	166,983,379,394	4,220	ordinary	/api/metasub41
RefSeq (85k taxID; release 97; with coordinates)	1.7 Tbp	31	626,753,663,468	85,375	with k-mer positions	/api/refseq85_coord
SRA-Fungi	160 Tbp	31	264,681,928,748	121,900	ordinary	/api/sra_fungi
SRA-MetaGut	156 Tbp	31	750,598,764,890	241,384	ordinary	/api/sra_metagut
SRA-Metazoa (1,000 studies)	119 Tbp	31	625,622,500,650	67,390	ordinary	/api/sra_metazoa1k
SRA-Microbe	221 Tbp	31	101,733,896,434	446,506	ordinary	/api/sra_microbe
SRA-Mouse (Mus Muculus)	147 Tbp	31	139,978,792,470	57,938	ordinary	/api/sra_mus_muculus
Tara Oceans (genomes with coordinates)	61.9 Gbp	31	26,511,636,608	34,815	with k-mer positions	/api/tara_genomes_coord
Tara Oceans (scaffolds)	357 Gbp	31	121,058,940,696	318,205,057	ordinary	/api/tara_assemblies
UHGG All	710 Gbp	31	33,120,094,297	286,997	ordinary	/api/uhgg_all
UHGG Catalogue	11.2 Gbp	31	9,676,446,464	4,644	ordinary	/api/uhgg

Supplementary Figure S-12: List of indexes available on MetaGraph Online. – Web view of indexes hosted on the MetaGraph Online search engine for sequence search.

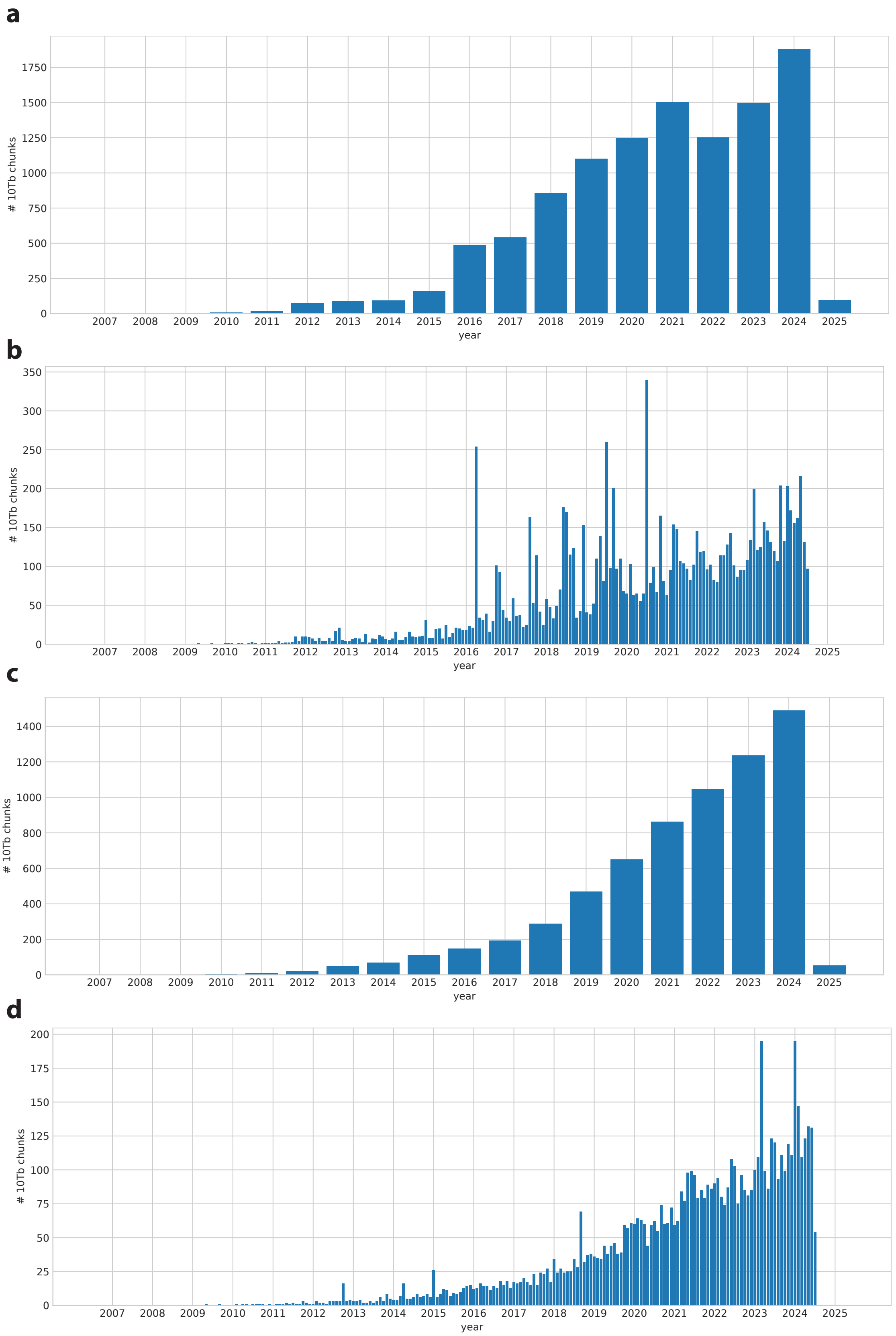

Supplementary Figure S-13: Annual growth of SRA. – We show the size increase of SRA over time using units of 10 Terabasepair (Tbp) chunks. Each chunk has the same size as our random subset used for cost extrapolation. Numbers for 2025 are lower, as the metadata forming the basis of this analysis has been collected on January 11th, 2025. **a)** Number of 10 Tbp chunks added per year. **b)** Number of 10 Tbp chunks added per month. **c)** Number of 10 Tbp chunks of public data added per year. **d)** Number of 10 Tbp chunks of public data added per month.

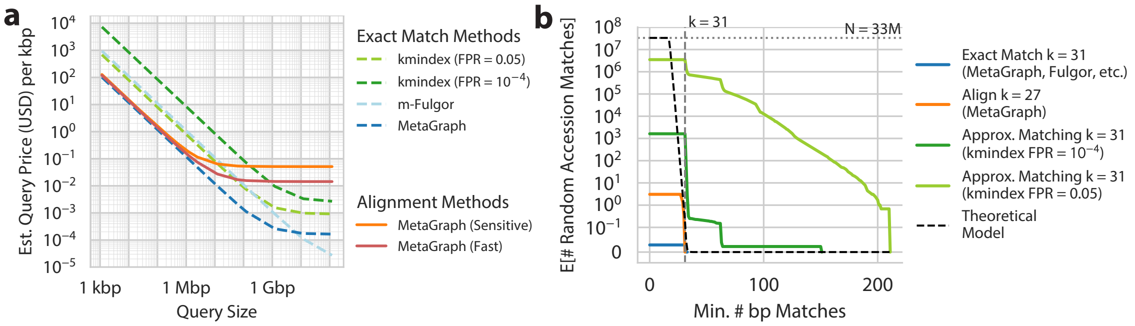

Supplementary Figure S-14: Estimated query cost and accuracy of MetaGraph indexes of the SRA. – **a)** Cloud compute costs for sequence queries of increasing size estimated by mapping 18,889 reads of length 100–250 randomly selected from the SRA-Public (100 studies) data against an index of the same data, and then scaled up to the whole set of publicly available sequences (as of January 11, 2025). For comparison, we have plotted the expected costs of meta-Fulgor[100] and kmindex[101]. For all methods, we assume that query throughput scales linearly with the number of available physical cores. **b)** Expected numbers of random matches of a sequence of length 100–250bp to the whole set of public sequences (as of January 11, 2025) reported by different. The dotted horizontal line represents the total number of public SRA accessions, while the dashed vertical line represents the k-mer size (31) for exact matching. Approximate matching was performed by kmindex[101], while exact matching and sequence-to-graph alignment were performed by MetaGraph. To store an index with a false positive rate of 0.05, kmindex needed 157 GB, while a false positive rate of 10^{-4} needed 1.1 TB. In contrast, the MetaGraph index requires only 51 GB. The "Theoretical Model" curve represents the theoretically expected number of matches of a random string of length 130 bp to the SRA (y-axis) where at least one k-mer of a given length (x-axis) is found exactly.

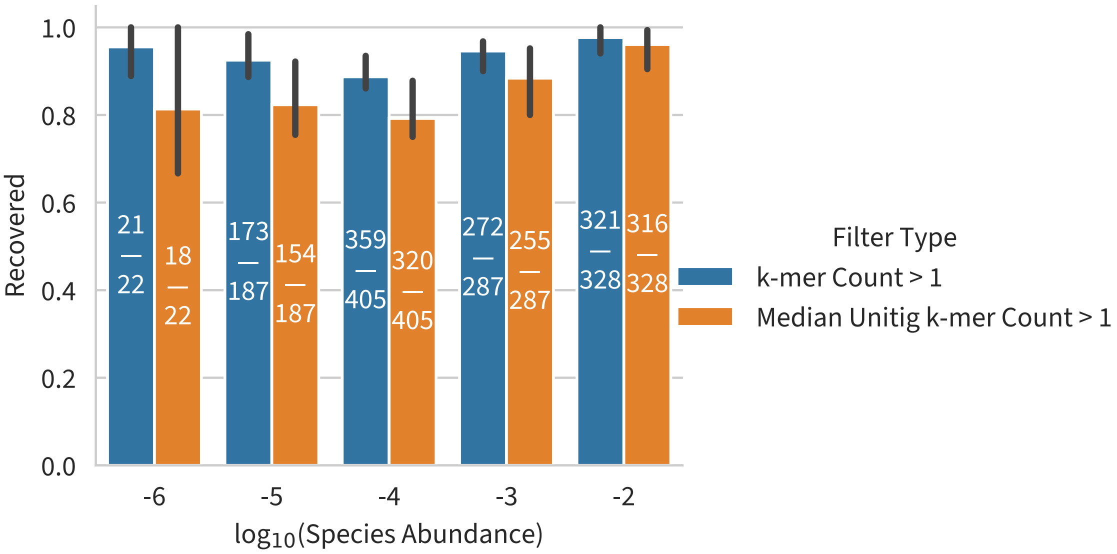

Supplementary Figure S-15: Realignability of reads to the MetaSUB graph using a simple singleton filtering strategy (blue) and our unitig-based cleaning strategy (orange) for species at different abundances in their respective original samples. Bars represent the mean recovery percentage from 1000 bootstrap samples of the reads, with error bars representing the 95% confidence intervals of the means. The observed numbers of reads where a species could be determined (correct species recovered / total) are overlaid on the bars.

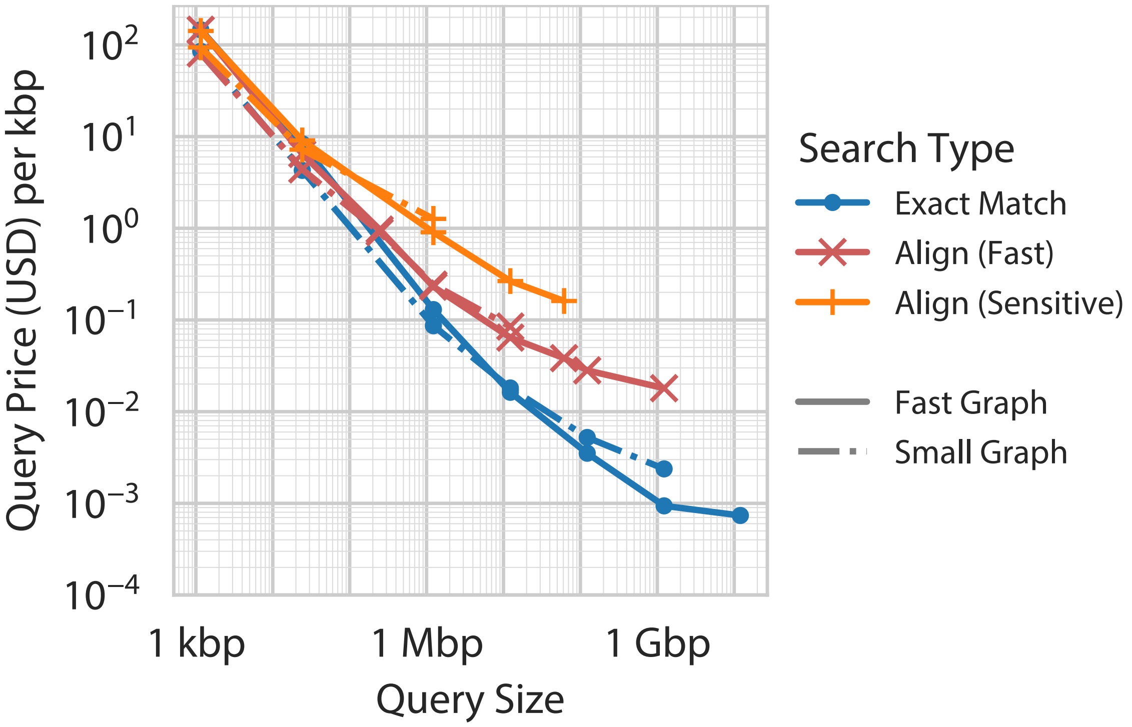

Supplementary Figure S-16: Query costs of MetaGraph indexes of the SRA. – Cloud compute costs for sequence queries of increasing size calculated by mapping query sets with reads of length 100–250 randomly selected from the SRA-Public (100 studies) data against 47 100-random-study indexes of Logan contigs[102], and then scaled up to the whole set of publicly available sequences (as of January 11, 2025).

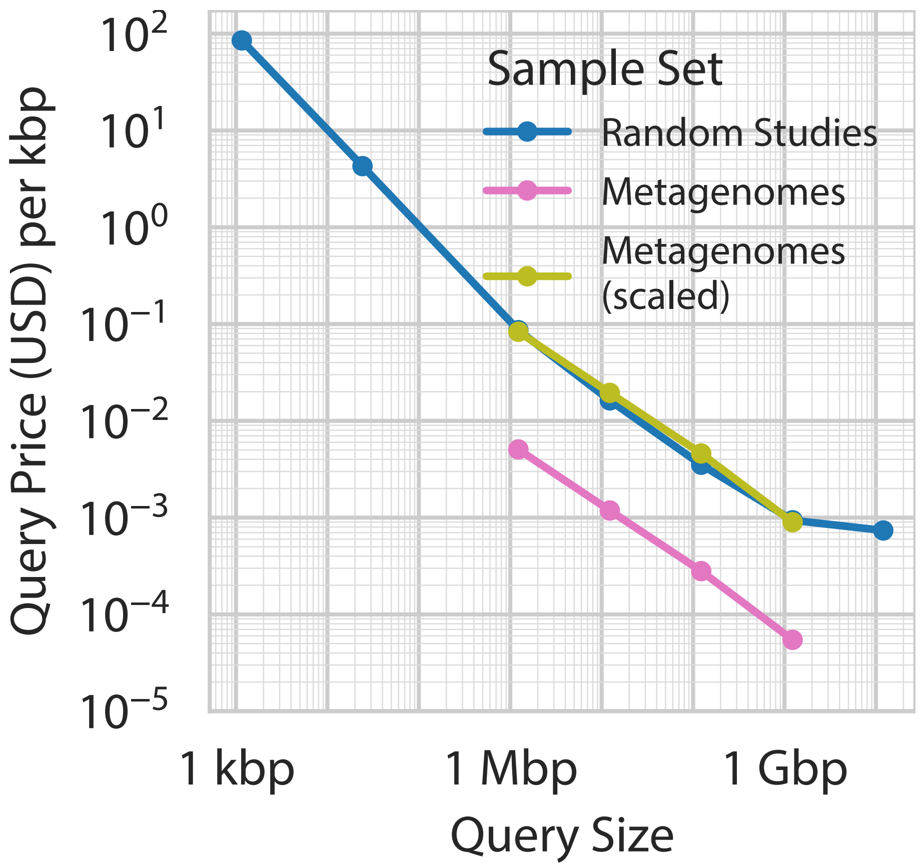

Supplementary Figure S-17: Query costs of MetaGraph metagenome indexes of the SRA. – Cloud compute costs for sequence queries of increasing size calculated by mapping query sets with reads of length 100–250 randomly selected from the SRA-Public (100 studies) data against up to 400 index chunks of randomly-selected metagenomes, and then scaled up to both the whole set of publicly available metagenome sequences (in pink), and the whole set of publicly available sequences (as of January 11, 2025; in green). The blue line shows our empirical query cost on the random studies chunks as a comparison.

Chapter C

Supplementary Tables

Supplementary Table S-3: Composition of the 5,184 samples of 100 random public studies from the SRA compared to a snapshot from June 6, 2024, the year the subset was taken.

	100-study subset		SRA Public	
**Property**	# samples	# Mbp	# samples	# Mbp
Total Size	5184	9,592,082	29,938,113	56,956,593,810
WGS	19.0%	32.5%	18.8%	36.4%
RNA-Seq	19.6%	46.5%	19.1%	36.9%
AMPLICON	20.4%	0.80%	44.0%	6.46%
OTHER	41.0%	20.1%	18.1%	20.2%
Illumina	90.3%	99.3%	89.7%	92.8%
454	4.8%	0.01%	1.23%	0.02%
Ion Torrent	4.2%	0.04%	1.52%	0.23%
PacBio	0.46%	0.60%	2.60%	1.63%
Other	0.24%	0.05%	4.95%	5.32%
Metagenome Amplicon	17.8%	0.55%	17.1%	0.68%
Whole Metagenomic Sequencing (WMGS)	2.2%	1.40%	2.54%	5.41%
Rest	80.0%	98.05%	80.36%	93.91%

Supplementary Table S-4: Indexing, hosting, and querying costs for a 100-study subset of the SRA, and extrapolations to the entire SRA. We assume an extrapolation factor of 6,986 based on the relative size difference between the 100-study subset and the public portion of the SRA as of January 11, 2025 (see **Table** S-3). See **Additional Resources Table 15** at `https://github.com/ratschlab/metagraph_paper_resources` for the formulas to calculate costs.

Sample Set	Property	Value
100-study	Construction time	16.4 h
	Number of Threads	34
	Peak RAM Usage	76 GB
	Cost	9.07 US$
	Index Size on Disk	51 GB
	Index Size in RAM	57 GB
	Compression	188 bp/byte
SRA Public (before 2025)	Preprocessing Cost	870,642 US$
	Indexing Cost	63,396 US$
	Index Storage Cost (per year)	89,779 US$
SRA Public (in 2024)	Preprocessing + Indexing Cost	215,927 US$

Supplementary References

[91] Gog, S., Beller, T., Moffat, A. & Petri, M. From theory to practice: Plug and play with succinct data structures. In *Lecture Notes in Computer Science (including subseries Lecture Notes in Artificial Intelligence and Lecture Notes in Bioinformatics)* (2014).

[92] Prezza, N. A Framework of Dynamic Data Structures for String Processing. In *International Symposium on Experimental Algorithms* (Leibniz International Proceedings in Informatics (LIPIcs), 2017).

[93] Conway, T. C. & Bromage, A. J. Succinct data structures for assembling large genomes. *Bioinformatics* **27**, 479–486 (2011). URL `https://academic.oup.com/bioinformatics/article-lookup/doi/10.1093/bioinformatics/btq697`.

[94] Bowe, A., Onodera, T., Sadakane, K. & Shibuya, T. Succinct de Bruijn graphs. In *Lecture Notes in Computer Science (including subseries Lecture Notes in Artificial Intelligence and Lecture Notes in Bioinformatics)* (2012).

[95] Karasikov, M. *et al.* Sparse Binary Relation Representations for Genome Graph Annotation. *Journal of Computational Biology* **27**, 626–639 (2020). URL `https://doi.org/10.1089/cmb.2019.0324`. PMID: 31891531, `https://doi.org/10.1089/cmb.2019.0324`.

[96] Muggli, M. D. *et al.* Succinct colored de Bruijn graphs. *Bioinformatics* **33**, 3181–3187 (2017). URL `https://doi.org/10.1093/bioinformatics/btx067`. `https://academic.oup.com/bioinformatics/article-pdf/33/20/3181/49042808/bioinformatics_33_20_3181.pdf`.

[97] Barbay, J., Claude, F. & Navarro, G. Compact binary relation representations with rich functionality. *Information and Computation* **232**, 19–37 (2013). URL `https://www.sciencedirect.com/science/article/pii/S0890540113001144`.

[98] Almodaresi, F., Pandey, P. & Patro, R. Rainbowfish: A Succinct Colored de Bruijn Graph Representation. In Schwartz, R. & Reinert, K. (eds.) *17th International Workshop on Algorithms in Bioinformatics (WABI 2017)*, vol. 88 of *Leibniz International Proceedings in Informatics (LIPIcs)*, 18:1–18:15 (Schloss Dagstuhl–Leibniz-Zentrum fuer Informatik, Dagstuhl, Germany, 2017). URL `http://drops.dagstuhl.de/opus/volltexte/2017/7657`.

[99] Bradley, P., den Bakker, H. C., Rocha, E. P., McVean, G. & Iqbal, Z. Ultrafast search of all deposited bacterial and viral genomic data. *Nature biotechnology* **37**, 152 (2019).

[100] Pibiri, G. E., Fan, J. & Patro, R. Meta-colored compacted de bruijn graphs. In Ma, J. (ed.) *Research in Computational Molecular Biology*, 131–146 (Springer Nature Switzerland, Cham, 2024).

[101] Lemane, T. *et al.* Indexing and real-time user-friendly queries in terabyte-sized complex genomic datasets with kmindex and ORA. *Nature Computational Science* **4**, 104–109 (2024).

[102] Chikhi, R., Raffestin, B., Korobeynikov, A., Edgar, R. & Babaian, A. Logan: Planetary-scale genome assembly surveys life's diversity. *bioRxiv* (2024). URL `https://www.biorxiv.org/content/early/2024/07/31/2024.07.30.605881`. `https://www.biorxiv.org/content/early/2024/07/31/2024.07.30.605881.full.pdf`.
