# Supplementary material for: Efficient and accurate search in petabase-scale sequence repositories
Source: Nature. 2025 Oct 8;647(8091):1036–44. doi: 10.1038/s41586-025-09603-w (PMC12657231; doi:10.1038/s41586-025-09603-w)
Supplement: Supplementary file 2 — Reporting Summary [file 41586_2025_9603_MOESM2_ESM.pdf]

nature portfolio

Corresponding author(s): Andre Kahles
Gunnar Raetsch

Last updated by author(s): Aug 20, 2025

Reporting Summary

Nature Portfolio wishes to improve the reproducibility of the work that we publish. This form provides structure for consistency and transparency in reporting. For further information on Nature Portfolio policies, see our Editorial Policies and the Editorial Policy Checklist.

Statistics

For all statistical analyses, confirm that the following items are present in the figure legend, table legend, main text, or Methods section.

n/a	Confirmed	
☐	☒	The exact sample size (*n*) for each experimental group/condition, given as a discrete number and unit of measurement
☒	☐	A statement on whether measurements were taken from distinct samples or whether the same sample was measured repeatedly
☐	☒	The statistical test(s) used AND whether they are one- or two-sided<br>*Only common tests should be described solely by name; describe more complex techniques in the Methods section.*
☐	☒	A description of all covariates tested
☐	☒	A description of any assumptions or corrections, such as tests of normality and adjustment for multiple comparisons
☐	☒	A full description of the statistical parameters including central tendency (e.g. means) or other basic estimates (e.g. regression coefficient) AND variation (e.g. standard deviation) or associated estimates of uncertainty (e.g. confidence intervals)
☐	☒	For null hypothesis testing, the test statistic (e.g. F, t, r) with confidence intervals, effect sizes, degrees of freedom and P value noted<br>*Give P values as exact values whenever suitable.*
☒	☐	For Bayesian analysis, information on the choice of priors and Markov chain Monte Carlo settings
☒	☐	For hierarchical and complex designs, identification of the appropriate level for tests and full reporting of outcomes
☒	☐	Estimates of effect sizes (e.g. Cohen's *d*, Pearson's *r*), indicating how they were calculated

Our web collection on statistics for biologists contains articles on many of the points above.

Software and code

Policy information about availability of computer code

Data collection

For data collection, we used the following software tools:
- sra_toolkit (v3.0.10)
- UCSC LiftOver tool (downloaded on 18.01.2021 from http://hgdownload.soe.ucsc.edu)
- KMC3 (https://github.com/karasikov/KMC; commit b163688)
- Google BigQuery (Service used; version N/A)
- ART (v2.5.8)
- pbsim (v3.0.0)

Data analysis

For the analysis of data, we have used the following software tools:
- Python (v3.9)-
- COBS (https://github.com/bingmann/cobs; commit 1cd6df2)
- kmindex (v0.5.3)
- Mantis (v0.2.0, https://github.com/splatlab/mantis; commit 0fb7dbb)
- Fulgor (v3.0.0)
- Themisto (v3.2.2)
- Bifrost (v1.3.5)
- BWA-MEM (v0.7.17-r1188)
- STAR (v2.7.0f)
- Snakemake (>= v5.0)
- GraphAligner (v1.0.17b)
- edlib (https://github.com/Martinsos/edlib; commit 931be2b)

- Mutation-Simulator (v3.0.1)
- Flask (v2.3.0)
- statsmodels (v0.14.0)
- scipy (v1.11.3)
- Nginx (v1.16.1)
- Docker (v1.13.1; API v1.26)
- PacBio CCS (v6.4.0)

All code for the MetaGraph framework and the accompanying analysis scripts is publicly available on GitHub;
https://github.com/ratschlab/metagraph; commit 30f6280
https://github.com/ratschlab/metagraph_paper_resources
https://github.com/ratschlab/metagraph-open-data

For manuscripts utilizing custom algorithms or software that are central to the research but not yet described in published literature, software must be made available to editors and reviewers. We strongly encourage code deposition in a community repository (e.g. GitHub). See the Nature Portfolio guidelines for submitting code & software for further information.

Data

Policy information about availability of data

All manuscripts must include a data availability statement. This statement should provide the following information, where applicable:

- Accession codes, unique identifiers, or web links for publicly available datasets
- A description of any restrictions on data availability
- For clinical datasets or third party data, please ensure that the statement adheres to our policy

Input data was collected from public sequence read archives, such as NCBI SRA, EBI ENA, or Genomic Data Commons using standard retrieval methods such as the sra toolkit or wget. In addition, we used public data provided in AWS S3 storage via the Logan project.

Following data sources have been used:
- NCBI Sequence Read Archive/ENA (samples available on January 11, 2025)
- RefSeq (release 97)
- UniParc (version 2023_04)
- UHGG (Unified Human Gut Genome, v1.0)
- Tara Oceans (v1.0)
- CARD (Comprehensive Antibiotic Resistance Database, v3.2.7)
- GENCODE annotation (v38)
- hg38 human reference genome (GENCODE v38)
- CHM13 human reference genome (v2.0)
- IsoCirc dataset (circular RNAs, GEO accession GSE141693)
- GTEx (Genotype Tissue Expression project, dbGaP phs000424.v7.p1)

Indexes of public sequence data are available at S3 Bucket s3://metagraph, also accessible via https://metagraph.s3.amazonaws.com/index.html. Further details and instructions for accessing the data can be found at https://github.com/ratschlab/metagraph-open-data. The web service is publicly available for web and API queries at https://metagraph.ethz.ch/search. Additional resources for this project, including sample metadata, interactive notebooks and analysis scripts are available in GitHub at https://github.com/ratschlab/metagraph_paper_resources. A list of all data sources and software used in context of this work is available as supplementary information.

Research involving human participants, their data, or biological material

Policy information about studies with human participants or human data. See also policy information about sex, gender (identity/presentation), and sexual orientation and race, ethnicity and racism.

Reporting on sex and gender	n/a
Reporting on race, ethnicity, or other socially relevant groupings	n/a
Population characteristics	n/a
Recruitment	n/a
Ethics oversight	n/a

Note that full information on the approval of the study protocol must also be provided in the manuscript.

Field-specific reporting

Please select the one below that is the best fit for your research. If you are not sure, read the appropriate sections before making your selection.

☒ Life sciences ☐ Behavioural & social sciences ☐ Ecological, evolutionary & environmental sciences

For a reference copy of the document with all sections, see nature.com/documents/nr-reporting-summary-flat.pdf

Life sciences study design

All studies must disclose on these points even when the disclosure is negative.

Sample size	The goal of our study was to build an index for biological sequencing samples at the petabase scale. For the SRA cohorts, we have selected all samples that were publicly available at the time of data access. For other cohorts (TCGA, GTEx, etc) we have included all available samples. That is, sample size was only limited through technical factors (e.g., availability for download).
Data exclusions	We have selected data to be indexed based on a pre-determined set of metadata values. For instance, data originating from 3rd generation long-read sequencing technologies has been excluded from parts of our study, as the error correction quality was not sufficient. All inclusion and exclusion criteria are clearly stated in the text. As the main point of our paper is that of scalability, the exclusion of a subset of inputs does not invalidate our findings.
Replication	We have replicated the key points of our manuscript (compressability, scalability) across 8 different input cohorts (all data is summarised in Tabl1 of the manuscript).
Randomization	For the extrapolation of index growth, we have subsampled all available input data uniformly at random.
Blinding	This is not applicable to our study as no subjects were recruited and only publicly available data was downloaded either exhaustively (comprising the full data sets) or as a subset selected uniformly at random.

Reporting for specific materials, systems and methods

We require information from authors about some types of materials, experimental systems and methods used in many studies. Here, indicate whether each material, system or method listed is relevant to your study. If you are not sure if a list item applies to your research, read the appropriate section before selecting a response.

Materials & experimental systems

n/a	Involved in the study	
☒	☐	Antibodies
☒	☐	Eukaryotic cell lines
☒	☐	Palaeontology and archaeology
☒	☐	Animals and other organisms
☒	☐	Clinical data
☒	☐	Dual use research of concern
☒	☐	Plants

Methods

n/a	Involved in the study	
☒	☐	ChIP-seq
☒	☐	Flow cytometry
☒	☐	MRI-based neuroimaging

Plants

Seed stocks	n/a
Novel plant genotypes	n/a
Authentication	n/a
