# Supplementary material for: Efficient and accurate search in petabase-scale sequence repositories
Source: Nature. 2025 Oct 8;647(8091):1036–44. doi: 10.1038/s41586-025-09603-w (PMC12657231; doi:10.1038/s41586-025-09603-w)
Supplement: Supplementary file 4 — A detailed listing of all software and databases (including versions/access dates) that were used for the presented work [file 41586_2025_9603_MOESM4_ESM.docx]

## **Software and Databases used**

| **Category** | **Software / Tool** | **Purpose in paper** | **Version as stated** | **Where stated** |
| --- | --- | --- | --- | --- |
| k-mer indexing, query experiments | **MetaGraph** | Used for experiments throughout the paper | <https://github.com/ratschlab/metagraph>; commit 30f6280 | Code Availability |
| Workflow engine | **Snakemake** | Workflows for index construction | **≥ 5** |  |
| Web backend | **Flask** | MetaGraph Online backend | **v2.3.0** | Methods (The architecture and the implementation of MetaGraph Online) |
| Web server | **Nginx** | Serves MetaGraph Online | **v1.16.1** |  |
| Container runtime | **Docker** | Deployment of web/backend + servers | **v1.13.1; API v1.26** |  |
| Language | **Python** | API client, orchestration | **v3.9** |  |
| Data query service | **Google BigQuery** | Producing SRA sample lists | Service used; version N/A | Data Availability mentions details in open‑data repo. |
| k-mer indexing | **COBS** | A Bloom filter-based competitor | <https://github.com/bingmann/cobs>; commit 1cd6df2 | Methods (Benchmarking COBS and kmindex) |
|  | **kmindex** | A Bloom filter-based competitor | **v0.5.3** |  |
|  | **Themisto** | A SBWT-based competitor | **v3.2.2** | Methods (Benchmarking Mantis, Themisto, Bifrost, and Fulgor) |
|  | **Mantis** | A CQF-based competitor | **v0.2.0 (**[https://github.com/splatlab/mantis](https://github.com/splatlab/mantis;l); commit 0fb7dbb**)** |  |
|  | **Bifrost** | A competing tool | **v1.3.5** |  |
|  | **Fulgor** | An SSHash-based competitor | **v3.0.0** |  |
| k-mer counting | **KMC3** | Counting k-mers before MetaGraph indexing | https://github.com/karasikov/KMC**;** commit b163688 | Methods (Scalable k-mer enumeration and counting) |
| AMR reference DB | **CARD** | Resistome analyses | **v3.2.7** | Methods (Human gut resistome and phageome exploration) |
| Statistical modelling and significance testing | **statsmodels** | Python package for binomial GLM fitting | **v0.14.0** |  |
| *p*-value correction | **scipy** | Python package for p-value adjustment | **v1.11.3** |  |
| DNA sequencing read sets | **Sequence Read Archive (SRA)** | Input data for indexing | **11.01.2025** | Introduction |
|  | **MetaSUB** |  | **Pilot dataset** | Methods (Indexing environmental metagenome samples (MetaSUB)) |
| Reference genomes | **RefSeq** |  | **Release 97** | Methods (Indexing the Reference Sequence (RefSeq) and UniParc collections) |
| Amino acid sequences | **UniParc** |  | **2023_04** |  |
| RNA-Seq data | **GTEx (Genotype Tissue Expression project)** |  | **dbGaP** phs000424.v7.p1 | Acknowledgements |
|  | **TCGA (The Cancer Genome Atlas project)** |  | **dbGaP** phs000178.v1.p1 |  |
| Assembled contigs | **Logan** |  | **v1.0** | Methods (Indexing a subset of the Logan dataset) |
|  | **UHGG (Unified Human Gut Genome)** |  | **v1.0** | Methods (Indexing the Unified Human Gastrointestinal Genome (UHGG)) |
|  | **Tara Oceans** |  | **v1.0** | Methods (Indexing global ocean microbiome (Tara Oceans) data) |
| Nucleotide mutation simulation | **Mutation‑Simulator** | Simulated mutations in reads | **v3.0.1** | Methods (Experiment discovery on SRA graphs) |
| Circular RNAs | **IsoCirc** |  | **GEO accession** GSE141693 | Methods (Survey of Back-splice Junctions) |
| Reference genome | **hg38** |  | **GRCh38.p13** (packaged with GENCODE v38) |  |
| Data analysis (alignment) | **BWA‑MEM** | Short‑read alignment in BSJ survey | **0.7.17‑r1188** |  |
| Data analysis (alignment) | **STAR** | RNA‑seq alignment in BSJ survey | **2.7.0f** |  |
| Reference annotation | **GENCODE** | Transcript annotation for BSJ analysis | **v38** |  |
| Coordinate mapping | **UCSC liftOver** | Genome coordinate conversion | **18.01.2021** (version not specified) |  |
| Distance/ED computation | **edlib** | Gold‑standard edit distances in Extended Data | <https://github.com/Martinsos/edlib>;  Commit 931be2b | Extended Data Fig. 2 caption. |
| Distance/ED computation | **GraphAligner** | Sequence-to-graph aligner | **v1.0.17b** |  |
| Reference genome | **CHM13** | Used as reference genome for read simulation in alignment accuracy study | **v2.0** |  |
| Sequencing read simulation | **ART** | Simulating sequencing reads for evaluating alignment accuracy. | **v2.5.8** |  |
|  | **pbsim** |  | **v3.0.0** |  |
|  | **PacBio CCS** |  | **v6.4.0** |  |
